# Are i‐Motif Structures in DNA Compatible with Cytosine–Ag(I)–Cytosine Base Pairs?

**DOI:** 10.1002/anie.202519809

**Published:** 2025-11-17

**Authors:** Victoria Seiffert, Carmen López‐Chamorro, Miquel Barceló‐Oliver, Catharina Erbacher, Uwe Karst, Marian Hebenbrock, Heinz‐Bernhard Kraatz, Miguel A. Galindo, Jens Müller

**Affiliations:** ^1^ Institut für Anorganische und Analytische Chemie Universität Münster Corrensstr. 28/30 48149 Münster Germany; ^2^ Departamento de Química Inorgánica Unidad de Excelencia Química Aplicada a Biomedicina y Medioambiente Facultad de Ciencias Universidad de Granada Avda Fuentenueva s/n Granada 18071 Spain; ^3^ Departament de Química Universitat de les Illes Balears carretera Valldemossa km 7.5, Ed. Mateu Orfila i Rotger Palma de Mallorca 07122 Spain; ^4^ Institut für Anorganische und Analytische Chemie Universität Münster Corrensstr. 48 48149 Münster Germany; ^5^ Department of Physical and Environmental Sciences University of Toronto Scarborough 1265 Military Trail Toronto M1C 1A4 Canada; ^6^ Center for Soft Nanoscience (SoN) and Cells in Motion Interfaculty Centre (CiMIC) Universität Münster Corrensstr. 28/30 48149 Münster Germany

**Keywords:** Bioinorganic chemistry, DNA, i‐motif, Metal‐mediated base pair

## Abstract

To evaluate whether the protons of hemi‐protonated C:CH^+^ base pairs in DNA i‐motif structures can be replaced by silver ions, an i‐motif/duplex junction (IDJ) known to be stable at pH 7 was investigated. Interestingly, only the most accessible out of five C:CH^+^ pairs in the structure can be converted into a C–Ag^I^–C base pair, as was derived by ^1^H NMR spectroscopy, luminescence spectroscopy, temperature‐dependent UV spectroscopy, and isothermal titration calorimetry (ITC). When more than one silver ion is present per IDJ, dynamic light scattering (DLS) and CD spectroscopy indicate that the IDJ rearranges into a larger aggregated structure, likely to contain non‐canonical silver‐mediated base pairs. The study suggests that—in contrast to earlier reports—the DNA i‐motif in general is not compatible with the formation of contiguous C–Ag^I^–C pairs.

## Graphical abstract

An i‐motif/duplex junction was evaluated with respect to its ability to replace the protons from the hemi‐protonated C:CH^+^ pairs by Ag^I^ ions to form C–Ag^I^–C base pairs. Interestingly, only one such metal‐mediated base pair can be formed before the oligonucleotide rearranges into a duplex structure. It was concluded that i‐motifs in general are *not* compatible with C–Ag^I^–C base pairs, in contrast to previous reports.

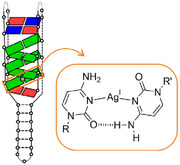

## Introduction

**Figure 1:**
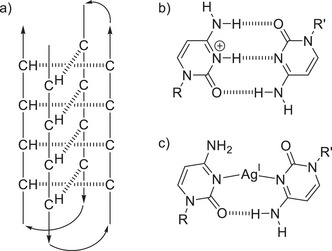
a) Schematic representation of an intramolecularly folded i‐motif structure. The arrows indicate the 5′→3′ direction of the oligonucleotide. Pairing patterns of b) hemi‐protonated C:CH^+^ and c) transoid C–Ag^I^–C base pairs. The latter contains one hydrogen bond only because the central N···N distance is much larger in N–Ag–N than in N–H···N, precluding the formation of a symmetric base pair^[^ ^18^, ^19^ ^]^ (R, R’ = DNA backbone).

DNA can adopt a variety of triplex and quadruplex structures distinct from the well‐known double‐helical B‐DNA.^[^ ^1^, ^2^, ^3^ ^]^ Among these, the i‐motif represents a quadruplex composed of two parallel‐stranded cytosine‐rich oligonucleotide duplexes intercalating in an antiparallel fashion (Figure 1a). Instead of the canonical base pairs, hemi‐protonated C:CH^+^ base pairs form a continuous stack in the center of this quadruplex (Figure 1b).^[^ ^1^, ^4^ ^]^ Due to the necessity of forming hemi‐protonated C:CH^+^ base pairs, the i‐motif structure is most stable under slightly acidic conditions (pH 4–6), correlating with the p*K*_a_ value of cytosine.^[^ ^5^, ^6^, ^7^, ^8^, ^9^ ^]^ Nevertheless, stable i‐motifs have also been observed under neutral conditions and even in vivo within the human genome.^[^ ^10^ ^]^ It has been noted that loops,^[^ ^11^, ^12^ ^]^ minor‐groove tetrads,^[^ ^13^, ^14^, ^15^ ^]^ and T:T mispairs can serve as stabilizing elements for i‐motif structures at pH 7.^[^ ^16^, ^17^ ^]^

An alternative approach to achieving stable i‐motif formation at neutral pH might involve the substitution of the proton of a hemi‐protonated C:CH^+^ pair by an Ag^I^ ion, leading to the formation of a metal‐mediated C–Ag^I^–C base pair. Such C–Ag^I^–C base pairs are well established in model complexes as well as in double‐helical DNA and RNA, and are known to enhance the thermal stability of a duplex.^[^ ^20^, ^21^, ^22^, ^23^, ^24^, ^25^, ^26^ ^]^ Figure 1c displays a C–Ag^I^–C pair with a transoid orientation of the glycosidic bonds, as geometrically imposed in an i‐motif structure. With an average Ag–N_pyrimidine_ bond length of 2.2 Å,^[^ ^27^ ^]^ it is not possible to form two hydrogen bonds next to a linear N–Ag^I^–N arrangement. Hence, this base pair is asymmetric and contains only one hydrogen bond.^[^ ^18^, ^19^ ^]^ A literature review shows that some reports exist on the formation of C–Ag^I^–C pairs within i‐motif structures.^[^ ^28^, ^29^, ^30^, ^31^ ^]^ However, other publications report the destabilization of the i‐motif structure in the presence of Ag^I^ and the formation of DNA duplexes composed of C–Ag^I^–C pairs instead.^[^ ^32^, ^33^, ^34^, ^35^ ^]^ In fact, the spectroscopic data used to deduce the formation of an Ag^I^‐containing i‐motif are likewise in agreement with C–Ag^I^–C base pair formation in a double helix (see Supporting Information for more details).

To reduce the probability that a cytosine‐rich oligonucleotide adopts a duplex structure in the presence of Ag^I^ ions, we evaluated a sequence known to fold into a stabilized i‐motif topology regarding its ability to incorporate Ag^I^ ions while retaining the tetra‐stranded structure. In particular, an i‐motif/duplex junction (IDJ) was investigated in which the i‐motif is stabilized by a minor‐groove tetrad, a T:T mispair, and an appended duplex.

## Results and Discussion

**Figure 2:**
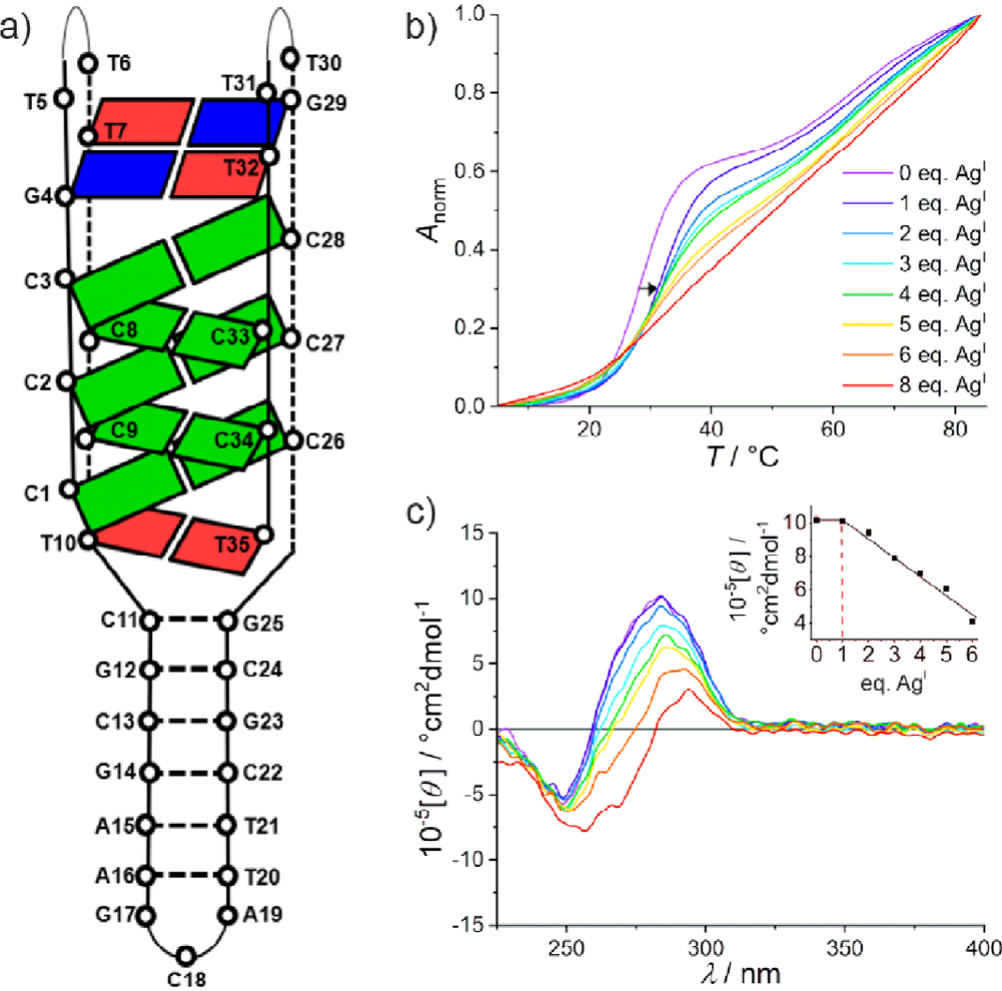
a) Schematic representation of IDJ1. Selected nucleobases are depicted as colored rectangles (C, green; T, red; G, blue). Redrawn from Ref. [36]. b) Melting curves and c) CD spectra of IDJ1 in the presence of increasing amounts of Ag^I^ (inset: molar ellipticity at 280 nm depending on the amount of Ag^I^). Conditions: 1 µm IDJ1, 50 mm NaClO_4_, 25 mm MOPS (pH 7.0).

The particular i‐motif‐duplex junction (IDJ1) had been reported to be stable at pH 7, and its structure had been determined by NMR spectroscopy.^[^ ^36^ ^]^ IDJ1 is composed of five C:CH^+^ base pairs (Figure 2a), in theory providing five potential high‐affinity binding sites for Ag^I^. Alternatively (or in addition), the canonical nucleobases may serve as Ag^I^‐binding sites, with the resulting structures being duplexes with Ag^I^‐mediated base pairs of (mostly) non‐Watson‐Crick‐type geometry.^[^ ^37^, ^38^, ^39^, ^40^, ^41^ ^]^ To evaluate the Ag^I^‐binding of IDJ1, Ag^I^ titration experiments were performed at pH 7, initially applying temperature‐dependent UV spectroscopy and CD spectroscopy. Spectra were recorded in the presence of increasing amounts of Ag^I^, aiming at a saturation of all potential Ag^I^‐binding sites of the i‐motif. Additionally, an excess of Ag^I^ was introduced to study possible effects.

The denaturation curve of IDJ1 reveals a biphasic melting behavior with *T*_m,1_ = 28.5 ± 0.1 °C and *T*_m,2_ = 64.7 ± 0.9 °C (Figure 2b). As elucidated previously,^[^ ^36^ ^]^ *T*_m,1_ corresponds to the melting of the quadruplex, whereas *T*_m,2_ is attributed to the unfolding of the hairpin. At first glance, the addition of 1 equiv of Ag^I^ results in an increase of the i‐motif melting temperature by Δ*T*_m,1_ = 3.2 ± 0.2 °C, with *T*_m,2_ remaining unaffected (Δ*T*_m,2_ = 1 ± 1 °C). This stabilization indicates the formation of one Ag^I^‐mediated base pair in the i‐motif structure (based on previous experiments with double‐stranded DNA showing that the formation of metal‐mediated base pairs typically leads to an increase in *T*_m_).^[^ ^22^ ^]^ Surprisingly, the addition of more than 1 equiv of Ag^I^ leads to a flattening of the melting curve. In the end, a biphasic melting behavior cannot be discerned anymore. This is indicative of substantial structural alterations.^[^ ^37^, ^38^, ^39^, ^40^, ^41^, ^42^, ^43^ ^]^ Taken together, the melting curves suggest that only one C–Ag^I^–C base pair is formed in the i‐motif. Rather than incorporating additional Ag^I^ ions into the i‐motif, the oligonucleotide appears to rearrange into a differently folded structure in the presence of more than 1 equiv of Ag^I^.

The CD spectrum of IDJ1 in the absence of Ag^I^ exhibits negative and positive Cotton effects at 250 and 284 nm, respectively, in agreement with the previous report (Figure 2c).^[^ ^36^ ^]^ The addition of 1 equiv of Ag^I^ does not have any noticeable influence on the CD spectrum, indicating that the proposed formation of the C–Ag^I^–C pair does not induce a relevant structural change. In contrast, the addition of larger amounts of Ag^I^ leads to a red‐shifted positive Cotton effect of reduced intensity, suggesting significant structural changes of IDJ1 as also proposed based on the melting curves.^[^ ^37^, ^38^, ^39^, ^40^, ^41^, ^42^, ^43^ ^]^ In fact, the CD spectrum in the presence of excess Ag^I^ is highly similar to the ones reported in previous publications on the purported incorporation of Ag^I^ ions into an i‐motif.^[^ ^28^, ^29^, ^31^ ^]^

**Figure 3:**
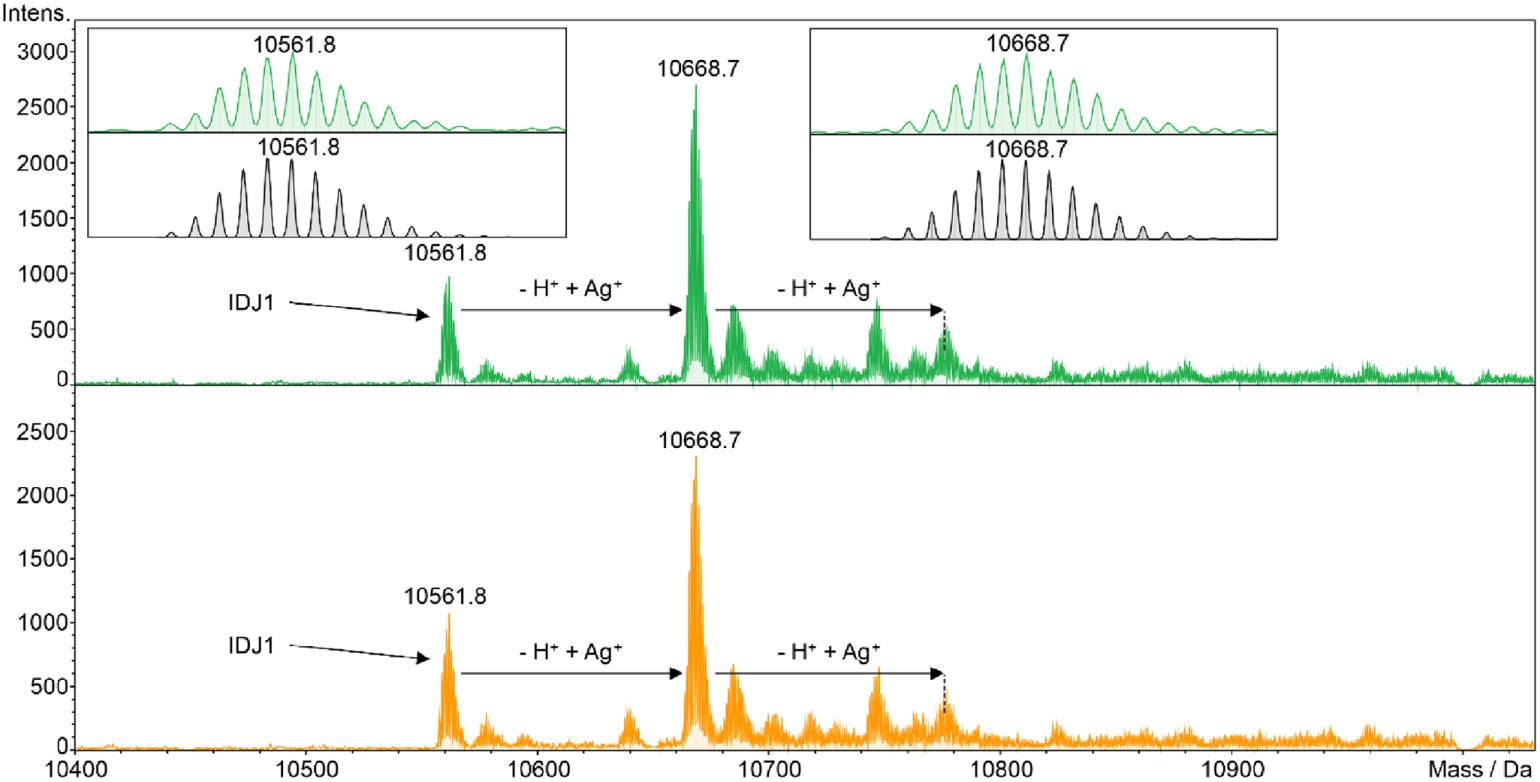
Deconvoluted ESI(–)‐MS spectra of IDJ1 in the presence of 2 (green) and 5 (orange) equiv of Ag^I^. The simulated isotopic patterns of the peaks of interest are shown as black insertions. Conditions: 5 µm IDJ1, 50 mm NaClO_4_, 25 mm MOPS (pH 7.0), 10 and 25 µm AgNO_3_, respectively, injected into an UltiMate 3000 UHPLC system upstream of the mass spectrometer. Mobile phase: aqueous 50 mm NH_4_OAc solution, flow rate: 0.4 mL min^−1^.

To confirm the stoichiometry of the Ag^I^:IDJ1 adduct, the Ag^I^‐binding was studied by electrospray ionization mass spectrometry (ESI‐MS). Figure 3 shows the deconvoluted mass spectra of IDJ1 recorded in the presence of 2 and 5 equiv of Ag^I^, respectively. In the spectra, the major peak is found at 10 668.7 Da, representing an adduct of IDJ1 and one Ag^I^ ion (calcd. 10 668.7 Da). The pure oligonucleotide IDJ1 gives rise to the additional minor peak at 10 561.8 Da (calcd. 10 561.8 Da). Despite the presence of excess Ag^I^, only one additional minor peak attributed to an adduct of IDJ1 and two Ag^I^ is observed, suggesting that it is caused by non‐specific binding of the second Ag^I^ ion. The mass spectra are therefore fully consistent with the formation of only one C–Ag^I^–C base pair in IDJ1. The larger aggregates proposed to form in the presence of excess Ag^I^ (based on the UV and CD spectroscopy data) are not detected in the mass spectrum because size exclusion chromatography was used to select the IDJ prior to ionization.

**Figure 4:**
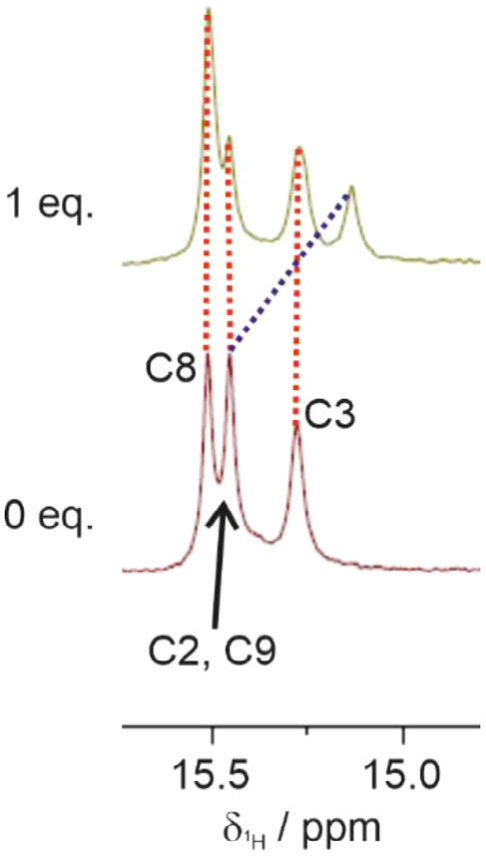
Excerpt of the ^1^H NMR spectrum of IDJ1 showing the imino resonances of the protonated cytosine moieties in the absence and presence of 1 equiv of Ag^I^. Dotted lines indicate the change in chemical shift upon Ag^I^ binding. A larger excerpt is shown in Figure S1. The peak assignment was taken from Ref. [36]. Please see Figure 2a for the residue numbering scheme. Conditions: 0.5 mm IDJ1, 50 mm NaClO_4_, 25 mm MOPS (pH 7.0), 9:1 H_2_O/D_2_O, 25 °C.

To gain insight into the location of the Ag^I^‐mediated base pair within IDJ1, ^1^H NMR spectroscopic studies were performed. As anticipated,^[^ ^36^ ^]^ the NMR spectrum of IDJ1 shows four resonances assigned to the imino protons of the protonated cytosine moieties within the i‐motif part of the structure (Figure 4). In agreement with the literature,^[^ ^36^ ^]^ the resonance of the imino proton of the C:CH^+^ pair next to the T:T mispair is not observed, likely due to rapid exchange with solvent protons. Upon the addition of 1 equiv of Ag^I^, one of the resonances experiences an upfield shift of 0.32 ppm (Figure 4, dotted blue line), all other resonances remain unaffected (Figure 4, red lines). As none of the four imino resonances disappears in the presence of Ag^I^, the C–Ag^I^–C pair cannot involve any of the corresponding cytosine residues. Hence, it is likely that the Ag^I^ ion inserts into the C:C pair adjacent to the T:T mispair, i.e., into the C1:C26 pair (Figure 2a). This assumption is corroborated by the observed upfield shift of the imino resonance of the neighboring C9:C34 pair.

**Figure 5:**
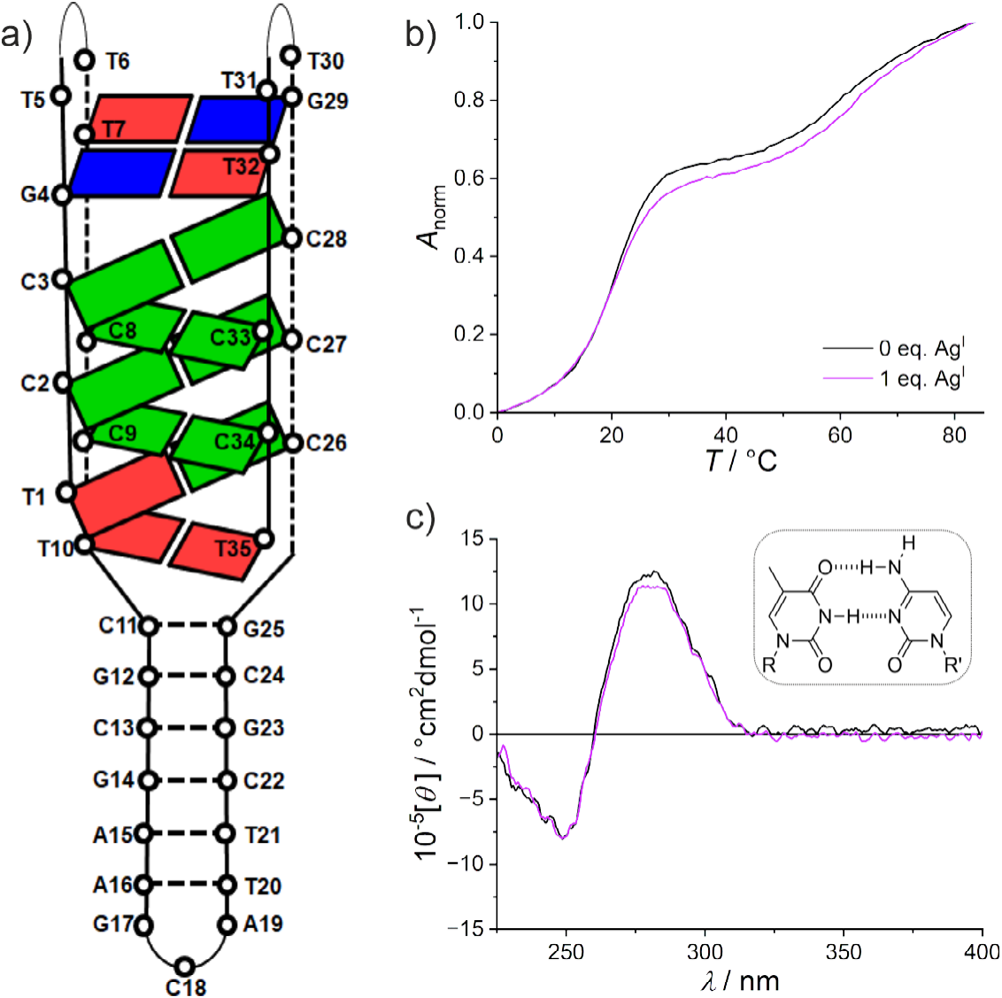
a) Schematic representation of IDJ1_T1. Selected nucleobases are depicted as colored rectangles (C, green; T, red; G, blue). b) Melting curves and c) CD spectra of IDJ1_T1 in the presence of increasing amounts of Ag^I^. The inset shows the structure of a T:C mismatch. Conditions: 1 µm IDJ1_T1, 50 mm NaClO_4_, 25 mm MOPS (pH 7.0).

For further confirmation of the site of metalation, several derivatives of IDJ1 were synthesized and investigated. In particular, selected individual cytosine residues were replaced by thymine or adenine moieties, leading to the presence of T:C and A:C mismatches, respectively. Ag^I^ titration experiments of the oligonucleotide IDJ1_T1, where the cytosine at position 1 is replaced by a thymine, reveal that the addition of 1 equiv of Ag^I^ does not lead to a significant change of *T*_m,1_ (Δ*T*_m,1_ = –0.4 ± 0.5 °C). Likewise, the CD spectrum does not change (Figure 5). Two conclusions can be drawn from this: 1) A metal‐mediated T–Ag^I^–C base pair is not formed.^[^ ^44^ ^]^ 2) The cytosine at position 1 in the unmodified IDJ1 is likely to be involved in C–Ag^I^–C base pair formation, as had already been predicted based on the ^1^H NMR spectra. Further modifications by replacing other cytosine residues with thymine support this conclusion (Figures S2–S8). For example, when C2, C3, C8, or C9 are substituted by thymine, i.e., cytosine residues not proposed to be involved in C–Ag^I^–C base pair formation, a stabilization of the i‐motif in the presence of 1 equiv of Ag^I^ is observed in the same order as for the unmodified IDJ1 (Table S1). In a few cases (i.e., when C27, C33, or C34 are replaced by thymine), the oligonucleotide structure is strongly destabilized in the absence *and* presence of Ag^I^, resulting in a large error in the determination of *T*_m_ and hence in a less reliable value for Δ*T*_m,1_. None of the CD spectra of IDJ1_T1 – IDJ1_T34 change upon the addition of 1 equiv of Ag^I^, which indicates that such a small amount of Ag^I^ does not suffice to initiate a rearrangement of these destabilized IDJs into a differently folded structure (Figures S2–S8). The IDJ modifications obtained when individual cytosine residues are replaced by adenine are strongly destabilized in the absence and presence of Ag^I^ (Table S1, Figures S9–S15), probably because the larger purine moiety distorts the IDJ structure. Notably, IDJ1_A1 is the most stable of these modifications and is not influenced by the presence of 1 equiv of Ag^I^, which is in agreement with C1:C26 being the site of metalation.

Several other modified or truncated versions of IDJ1 were investigated, too. IDJ2 lacks the T:T mispair acting as a stabilizing element for the i‐motif. While showing biphasic melting behavior, IDJ2 does not feature an increase in *T*_m,1_ in the presence of 1 equiv of Ag^I^ (Figure S16). It might therefore be assumed that the Ag^I^ ion in IDJ1 actually binds the T:T mispair, yielding a T–Ag^I^–T pair.^[^ ^41^ ^]^ To rule out this possibility, IDJ2 was further modified. The resulting IDJ3 contains one unpaired thymidine residue instead of the T:T mismatch (Figure S17). Here, the addition of 1 equiv of Ag^I^ induces a stabilization of the i‐motif by Δ*T*_m,1_ = 2.6 ± 0.4 °C. Hence, the T:T mismatch is not involved in the formation of the Ag^I^‐mediated base pair. This is further corroborated by the mass spectra shown in Figure 3, because a hypothetical T–Ag^I^–T pair would result in the loss of two protons and hence in a different molecular mass. Nevertheless, the presence of the T:T pair is required to obtain a sufficiently stable IDJ1. Similarly, omitting the entire duplex part (IDJ4, Figure S18) or the G:T:G:T tetrad (IDJ5, Figures S19–S21), both known to be relevant for the stabilization of IDJ1,^[^ ^45^ ^]^ leads to a large destabilization of the i‐motif. The resulting structures are not stabilized upon the addition of 1 equiv of Ag^I^ (Table S1), further indicating the necessity of a stabilized i‐motif for C–Ag^I^–C pair formation.

In a different experiment, the C:CH^+^ pair composed of C1 and C26, proposed to be the residues involved in Ag^I^‐mediated base pair formation, was replaced by a T:T mispair (IDJ6, Figure S22). Not unexpectedly, the presence of 1 equiv of Ag^I^ does not lead to a change of *T*_m,1_, further supporting the notion that the C–Ag^I^–C base pair in IDJ1 involves C1 and C26.

**Figure 6:**
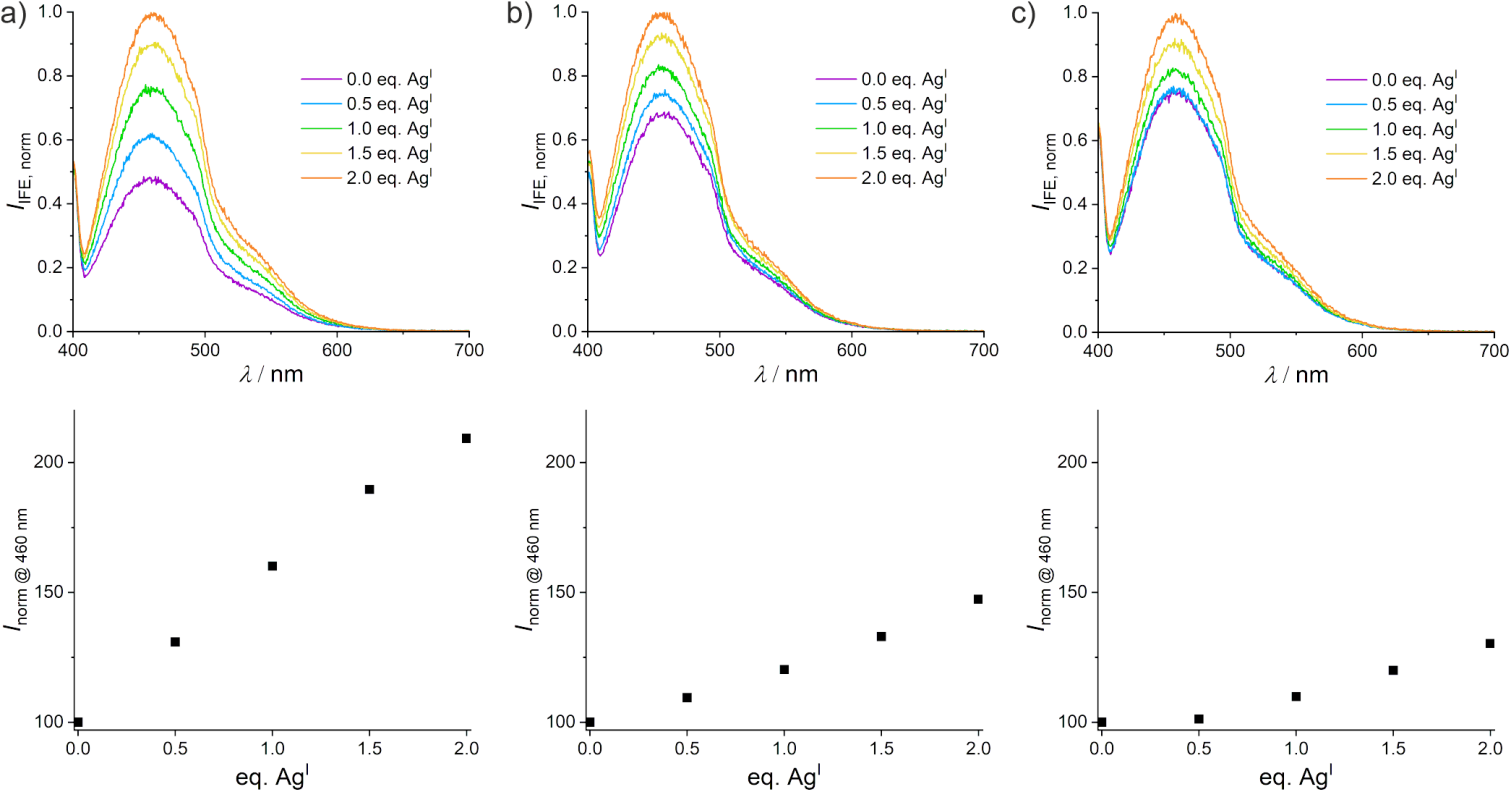
Normalized luminescence spectra (top) and relative change in luminescence at 460 nm (bottom) for a) IDJ1_P1, b) IDJ1_P8, and c) IDJ1_P27 in the presence of increasing amounts of Ag^I^. Conditions: 1 µm oligonucleotide, 50 mm NaClO_4_, 25 mm MOPS (pH 7.0), *λ*_exc_ = 350 nm.

To collect additional evidence for the formation of the C–Ag^I^–C base pair at the edge of the i‐motif, the fluorescent cytosine analogue pyrrolocytosine (P) was introduced at positions 1, 8, and 27, respectively. The luminescence of this nucleobase is known to change upon its incorporation into a Ag^I^‐mediated base pair.^[^ ^46^, ^47^ ^]^ The melting curves of the resulting three derivatives IDJ1_P1, IDJ1_P8, and IDJ1_P27 exhibit the anticipated biphasic melting behavior (Figures S23–S25, Table S1). Upon the addition of 1 equiv of Ag^I^, *T*_m,1_ increases slightly but significantly in all three cases, resembling the observation made with the unmodified IDJ1. It can therefore be concluded that the three fluorescent derivatives are also capable of forming one Ag^I^‐mediated base pair in the i‐motif. The CD spectra show, both in the absence and in the presence of 1 equiv of Ag^I^, the expected Cotton effects at around 250 nm (negative) and 284 nm (positive), typical for the IDJ,^[^ ^36^ ^]^ indicating no significant structural changes upon the incorporation of pyrrolocytosine. Figure 6 depicts the luminescence at 460 nm of IDJ1_P1, IDJ1_P8, and PDJ1_P27 in the presence of increasing amounts of Ag^I^. In all cases, the luminescence increases linearly upon the addition of Ag^I^. This may sound surprising at first glance, because previous reports on P–Ag^I^–C formation had shown a decrease in luminescence upon Ag^I^ binding.^[^ ^46^, ^47^ ^]^ However, those studies involved nucleic acid duplexes in which the P residue was extruded from the helix because of the unstable P:C pair in the absence of Ag^I^. Luminescence decrease was then caused by the incorporation of P into the base stack upon P–Ag^I^–C pair formation. In contrast, stable P:CH^+^ pairs and hence P moieties included in the base stack are present in the IDJs even in the absence of Ag^I^. As a result, the increase in luminescence upon the transition from a P:CH^+^ pair to a P–Ag^I^–C pair likely indicates a local rigidification of the structure. It should be noted that a precedent exists for an increase in luminescence upon Ag^I^‐mediated base pair formation.^[^ ^48^ ^]^ In the present study, the increase observed for IDJ1_P1 is more pronounced than that observed for IDJ1_P8 and IDJ1_P27. Hence, the formation of the Ag^I^‐mediated base pair has the most profound influence on the emission intensity when the luminescent cytosine analogue is located at position 1. This observation supports the proposal that the nucleobases at positions 1 and 26 are involved in the C–Ag^I^–C base pair formation. The farther away the pyrrolocytosine is located from positions 1 and 26, the less pronounced are the changes in luminescence intensity upon the addition of Ag^I^. The fact that the luminescence intensity continues to increase in the presence of more than 1 equiv of Ag^I^ suggests that the P–Ag^I^–C pairs are labile. Hence, according to Le Chatelier's principle, larger amounts of Ag^I^ drive the equilibrium to completeness and lead to an on average more rigid structure.

Isothermal titration calorimetry (ITC) was performed in an attempt to quantify the interaction between IDJ1 and Ag^I^ ions and to confirm the stoichiometry of the adduct. The experiments revealed a weak interaction between Ag^I^ and IDJ1, with an approximate stoichiometry of 1:1 and a dissociation constant of about 10^−4^ M. This results in a *c* value that is too low (approximately 0.5) for a precise determination of the thermodynamic parameters, considering the concentrations at which DNA can be worked with without aggregation. When the Ag^I^ concentration was increased to better understand the interaction, additional interactions were discovered, starting at four Ag^I^ ions per IDJ1. These observations essentially confirm that only one Ag^I^ ion can be inserted site‐specifically into the i‐motif structure of IDJ1. The addition of further Ag^I^ ions then initiates a structural rearrangement, likely giving rise to a fully metalated double‐helical structure composed of Ag^I^‐mediated base pairs.

Dynamic light scattering (DLS) was applied to investigate the Ag^I^ binding of IDJ1 even further. In particular, the hydrodynamic radii *r*_H_ of the particles in solution were determined. When estimating the theoretical hydrodynamic radius, a small oligonucleotide can, in principle, be modeled as a spherical particle or as a symmetrical cylinder. Based on the ratio *q* of the length *L* and the diameter *d* of the particle, it can be evaluated whether a spherical model (*q* < 2) or a cylinder model (2 < *q* < 30) is more appropriate.^[^ ^49^ ^]^ For IDJ1, *L* and *d* were estimated as 5.62 and 2.5 nm, respectively, based on the published solution structure.^[^ ^36^ ^]^ Hence, a cylinder model was applied, giving an estimated *r*_H_ of 2.08 nm (see Supporting Information for details regarding the calculation). Based on the DLS measurements (Figure S26), the experimental hydrodynamic radius *r*_H_ amounts to 2.09 nm prior to the addition of Ag^I^, matching the theoretical value. In the presence of 1 equiv of Ag^I^, *r*_H_ remains exactly the same, indicating no structural change. In contrast, two species are detected in solution when excess (i.e., 5 equiv) Ag^I^ is present. These two species have hydrodynamic radii of 2.09 and 70.9 nm, respectively, showing that the original (likely mono‐metalated) IDJ1 is still present but that an additional, much larger aggregate has formed. Based on the *r*_H_ of 70.9 nm, the latter could be, for example, a spherical particle of ca. 70 nm radius or a much longer symmetrical cylinder. This finding is in agreement with a structural reorganization toward a larger, differently folded structure in the presence of excess Ag^I^, as had been proposed based on the flattening of the melting curves and the substantial changes in the CD spectrum.

## Conclusion

**Figure 7:**
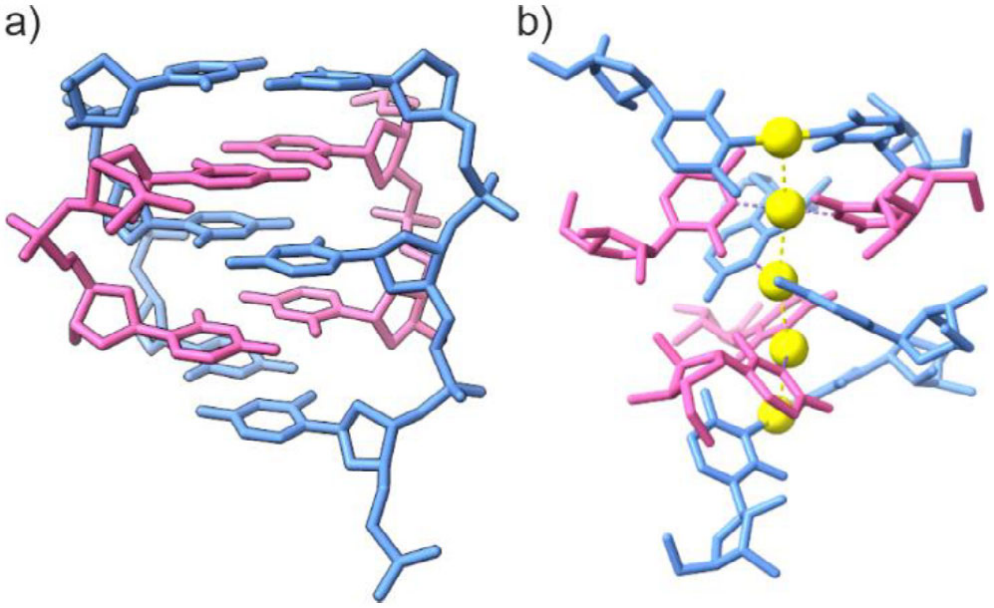
Experimental structures of a) the i‐motif section of IDJ1 (PDB 7O5E)^[^ ^36^ ^]^ and b) [Ag(cytidine‐*N3*)_2_]NO_3_ (CCDC 1861420, depiction of five formula entities, anions not shown).^[^ ^52^ ^]^

We were able to show that the proton of a hemi‐protonated C:CH^+^ pair in an i‐motif can, in principle, be replaced by an Ag^I^ ion, giving rise to the formation of a C–Ag^I^–C base pair. However, it is important that several stabilizing moieties are present such as an adjacent T:T mispair, a minor‐groove tetrad, and an appended hairpin duplex. Ag^I^ titration experiments followed by temperature‐dependent UV spectroscopy, by CD spectroscopy, by mass spectrometry, by ^1^H NMR spectroscopy, and by isothermal titration calorimetry indicate that the i‐motif/duplex junction under investigation forms only a single C–Ag^I^–C base pair, even though up to five Ag^I^ insertions could have been expected based on the oligonucleotide sequence. Using ^1^H NMR spectroscopy and titrations with appropriately modified oligonucleotides, the C–Ag^I^–C base pair was determined to be located at the terminus of the i‐motif structure, which—in agreement with NMR spectroscopic data—appears to be one of the more flexible parts of the structure.^[^ ^36^ ^]^ It can therefore be speculated that only the most accessible C:CH^+^ pair can be metalated to form a metal‐mediated base pair. In the presence of more than 1 equiv of Ag^I^, a rearrangement takes place toward differently folded structures. Based on the CD spectra of the latter, they are proposed to be duplexes comprising Ag^I^‐mediated base pairs of canonical nucleobases.^[^ ^40^ ^]^ The formation of a fully metalated i‐motif with all protons of the hemi‐protonated C:CH^+^ pairs replaced by Ag^I^ ions is ruled out for the sequence under investigation and is unlikely to exist for any other i‐motif. Previously reported i‐motifs with neighboring Ag^I^‐mediated base pairs are likely to have been misassigned as duplexes with C–Ag^I^–C base pairs.^[^ ^28^, ^29^, ^30^, ^31^ ^]^ In this context, it is interesting to note that an oligonucleotide sequence known to adopt an i‐motif structure in the absence of Ag^I [^ ^50^ ^]^ crystallizes in a non‐canonical structure bearing a DNA‐templated Ag nanocluster in the presence of Ag^I^,^[^ ^51^ ^]^ providing another example for the incompatibility of the i‐motif architecture with C–Ag^I^–C pairs. It can be speculated that the asymmetric transoid C–Ag^I^–C pair (Figure 1c)^[^ ^18^, ^19^ ^]^ does not fit into the rigid structure of an i‐motif (Figure 7a), so that cytosine‐rich oligonucleotides prefer to form duplexes with Ag^I^‐mediated base pairs instead, if excess Ag^I^ ions are available.^[^ ^32^ ^]^ In fact, the previously reported crystal structure of the metal complex [Ag(cytidine‐*N3*)_2_]NO_3_ shows a chain of closely spaced [Ag(cytidine‐*N3*)_2_]^+^ entities, assembled via hydrogen‐bonding, stacking, and argentophilic interactions (Figure 7b).^[^ ^52^ ^]^ This structure indicates the preferred geometrical arrangement of C–Ag^I^–C base pairs without additional constraints imposed by a DNA backbone. It differs significantly from the geometry imposed by an i‐motif (Figure 7a). Consistent with this line of argumentation, the more symmetric C–Cu^I^–C pair^[^ ^53^ ^]^ appears to be able to stabilize an i‐motif via Cu^I^‐mediated base pair formation.^[^ ^54^ ^]^ Our findings show that the repertoire of nucleic acid structures capable of forming well‐defined Ag^I^‐modified derivatives cannot be extended from duplexes^[^ ^22^ ^]^ and triplexes^[^ ^55^, ^56^, ^57^ ^]^ to i‐motifs.

## Supporting Information

The authors have cited additional references within the Supporting Information.^[^ ^58^, ^59^, ^60^, ^61^ ^]^

## Conflict of Interests

The authors declare no conflict of interest.

## Supporting information



Supporting Information

## Data Availability

The data that support the findings of this study are available from the corresponding author upon reasonable request.
