# Supplementary material for: Are i‐Motif Structures in DNA Compatible with Cytosine–Ag(I)–Cytosine Base Pairs?
Source: Angew Chem Int Ed Engl. 2025 Nov 17;65(2):e19809. doi: 10.1002/anie.202519809 (PMC12790311; doi:10.1002/anie.202519809)
Supplement: Supplementary file 1 — Supporting Information [file ANIE-65-e19809-s001.pdf]

Table of Contents

Fig. S1. Excerpt from the ^{1}H NMR spectrum of IDJ1 .... S2
Fig. S2. Melting curves and CD spectra of IDJ1_T2 .... S3
Fig. S3. Melting curves and CD spectra of IDJ1_T3 .... S3
Fig. S4. Melting curves and CD spectra of IDJ1_T8 .... S4
Fig. S5. Melting curves and CD spectra of IDJ1_T9 .... S4
Fig. S6. Melting curves and CD spectra of IDJ1_T27 .... S5
Fig. S7. Melting curves and CD spectra of IDJ1_T33 .... S5
Fig. S8. Melting curves and CD spectra of IDJ1_T34 .... S6
Fig. S9. Melting curves and CD spectra of IDJ1_A1 .... S7
Fig. S10. Melting curves and CD spectra of IDJ1_A2 .... S8
Fig. S11. Melting curves and CD spectra of IDJ1_A3 .... S9
Fig. S12. Melting curves and CD spectra of IDJ1_A8 .... S10
Fig. S13. Melting curves and CD spectra of IDJ1_A27 .... S11
Fig. S14. Melting curves and CD spectra of IDJ1_A33 .... S12
Fig. S15. Melting curves and CD spectra of IDJ1_A34 .... S13
Fig. S16. Melting curves and CD spectra of IDJ2 .... S14
Fig. S17. Melting curves and CD spectra of IDJ3 .... S14
Fig. S18. Melting curves and CD spectra of IDJ4 .... S15
Fig. S19. Melting curves and CD spectra of IDJ5 .... S15
Fig. S20. Melting curves and CD spectra of IDJ5_T1 .... S16
Fig. S21. Melting curves and CD spectra of IDJ5_T3 .... S16
Fig. S22. Melting curves and CD spectra of IDJ6 .... S17
Fig. S23. Melting curves and CD spectra of IDJ1_P1 .... S17
Fig. S24. Melting curves and CD spectra of IDJ1_P8 .... S18
Fig. S25. Melting curves and CD spectra of IDJ1_P27 .... S18
Fig. S26. Intensity plots of the DLS measurements S19
Fig. S27. HPLC chromatograms of the oligonucleotides S20
Table S1. Overview of $T_{m,1}$ and $\Delta T_{m,1}$ values S22
Experimental Details S23
Alternative explanation of the data from *Chem. Commun.* 2013, *49*, 7696 .... S26
Alternative explanation of the data from *Chem. Commun.* 2014, *50*, 15385 .... S26
Alternative explanation of the data from *ACS Sens.* 2020, *5*, 2177 .... S26
Alternative explanation of the data from *Angew. Chem. Int. Ed.* 2024, *63*, e202407838 .... S27
References S28

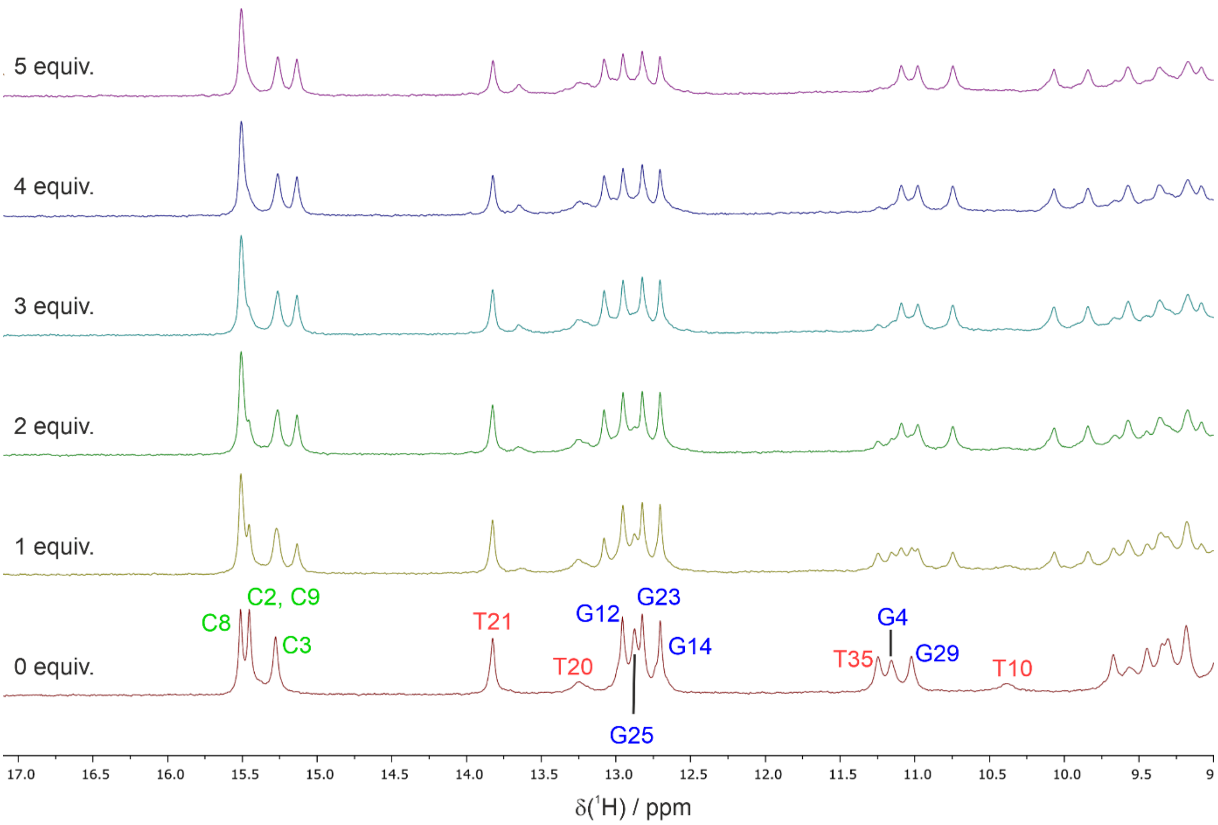

Figure S1. Excerpt from the ^{1}H NMR spectrum of IDJ1 showing the imino resonances in the presence of increasing amounts of Ag^{I}. The assignment of the resonances is based on a literature report (C, green; T, red; G, blue).[1] Major changes occur only upon the addition of the first equivalent of Ag^{I}. Relevant changes are observed in the region of the C:CH^{+} resonances (>15 ppm) and the resonance of the T:T mispair (originally at ca. 10.4 ppm), indicating structural changes in their direct vicinity. Conditions: 0.5 mM IDJ1, 50 mM $NaClO_4$, 25 mM MOPS (pH 7.0), 9:1 H_2O/D_2O, 25 °C.

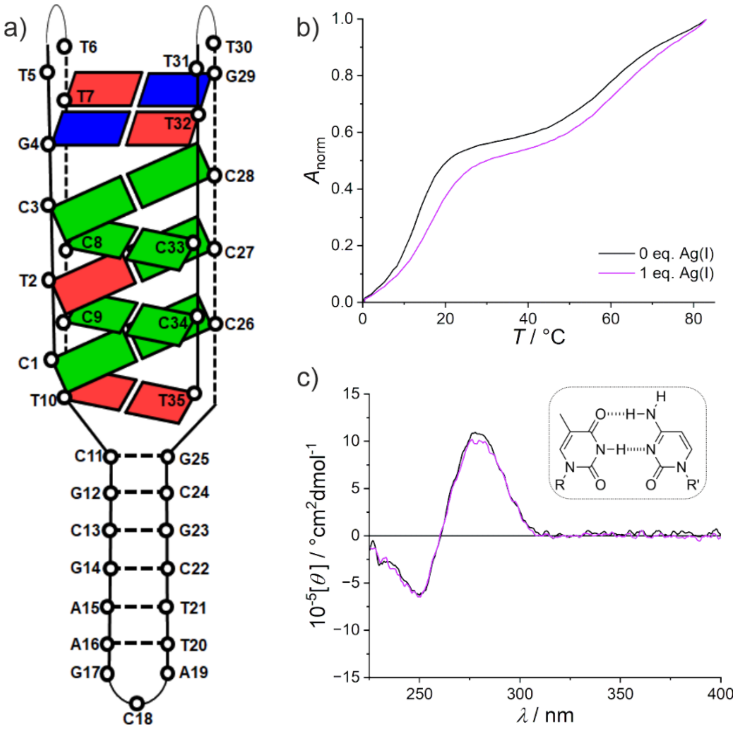

Figure S2. a) Schematic representation of IDJ1_T2. Selected nucleobases are depicted as colored rectangles (C, green; T, red; G, blue). b) Melting curves and c) CD spectra of IDJ1_T2 in the presence of increasing amounts of Ag^{I}. The inset shows the structure of a T:C mismatch. Conditions: 1 μM IDJ1_T2, 50 mM $NaClO_4$, 25 mM MOPS (pH 7.0).

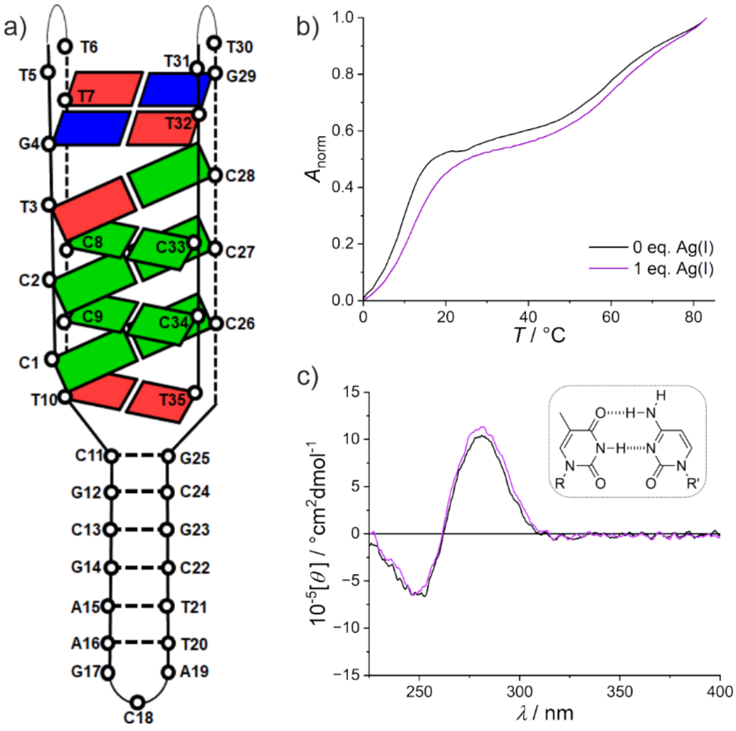

Figure S3. a) Schematic representation of IDJ1_T3. Selected nucleobases are depicted as colored rectangles (C, green; T, red; G, blue). b) Melting curves and c) CD spectra of IDJ1_T3 in the presence of increasing amounts of Ag^{I}. The inset shows the structure of a T:C mismatch. Conditions: 1 μM IDJ1_T3, 50 mM $NaClO_4$, 25 mM MOPS (pH 7.0).

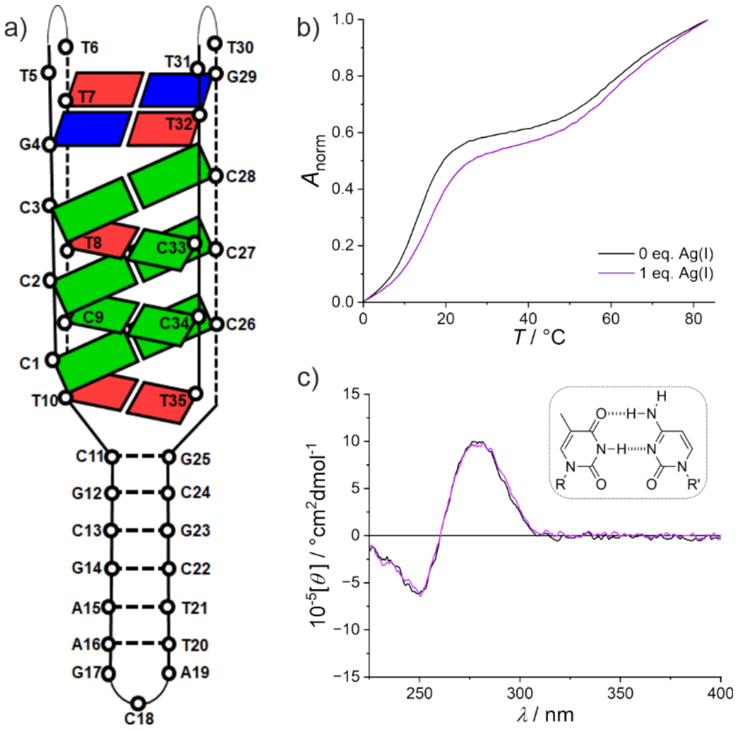

Figure S4. a) Schematic representation of IDJ1_T8. Selected nucleobases are depicted as colored rectangles (C, green; T, red; G, blue). b) Melting curves and c) CD spectra of IDJ1_T8 in the presence of increasing amounts of Ag^{I}. The inset shows the structure of a T:C mismatch. Conditions: 1 µM IDJ1_T8, 50 mM $NaClO_4$, 25 mM MOPS (pH 7.0).

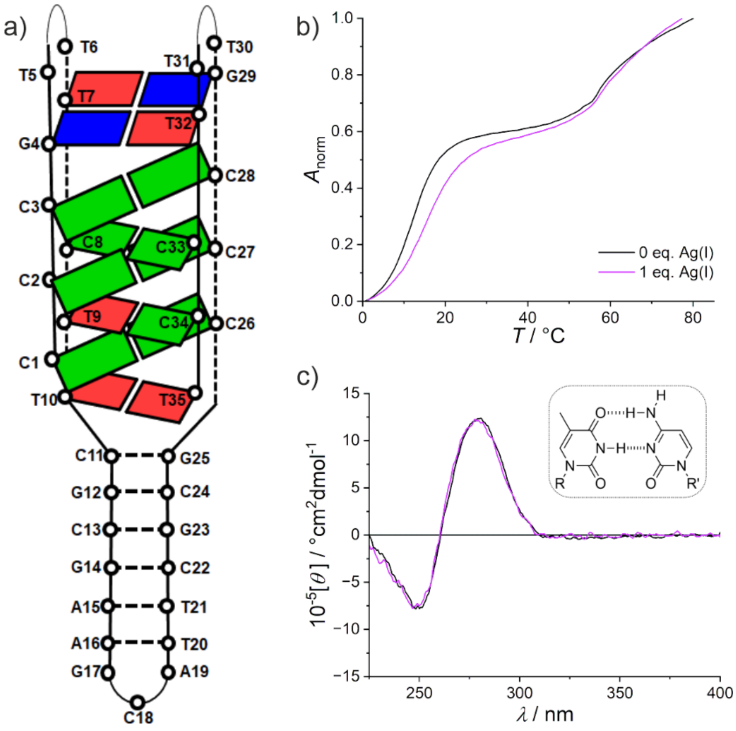

Figure S5. a) Schematic representation of IDJ1_T9. Selected nucleobases are depicted as colored rectangles (C, green; T, red; G, blue). b) Melting curves and c) CD spectra of IDJ1_T9 in the presence of increasing amounts of Ag^{I}. The inset shows the structure of a T:C mismatch. Conditions: 1 µM IDJ1_T9, 50 mM $NaClO_4$, 25 mM MOPS (pH 7.0).

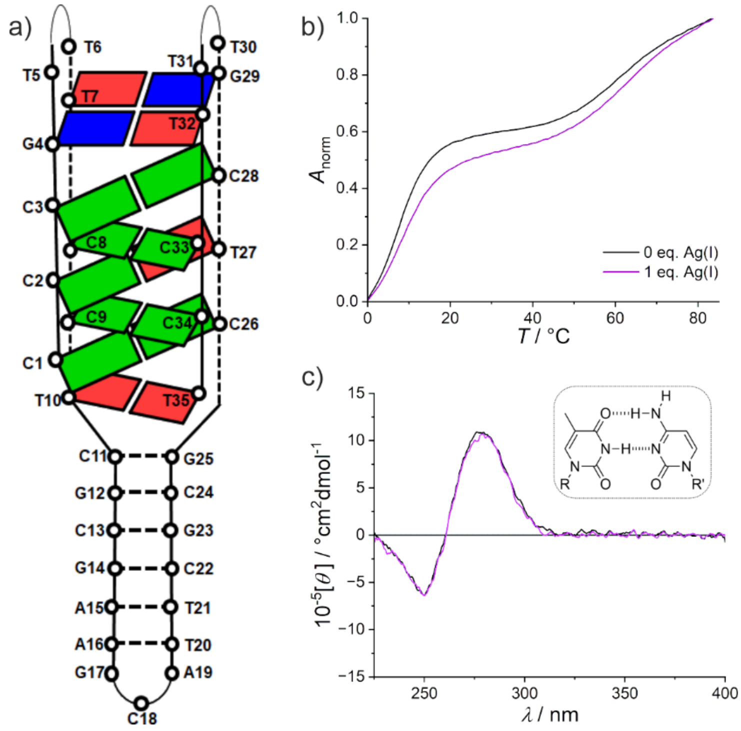

Figure S6. a) Schematic representation of IDJ1_T27. Selected nucleobases are depicted as colored rectangles (C, green; T, red; G, blue). b) Melting curves and c) CD spectra of IDJ1_T27 in the presence of increasing amounts of Ag^{I}. The inset shows the structure of a T:C mismatch. Conditions: 1 μM IDJ1_T27, 50 mM $NaClO_4$, 25 mM MOPS (pH 7.0).

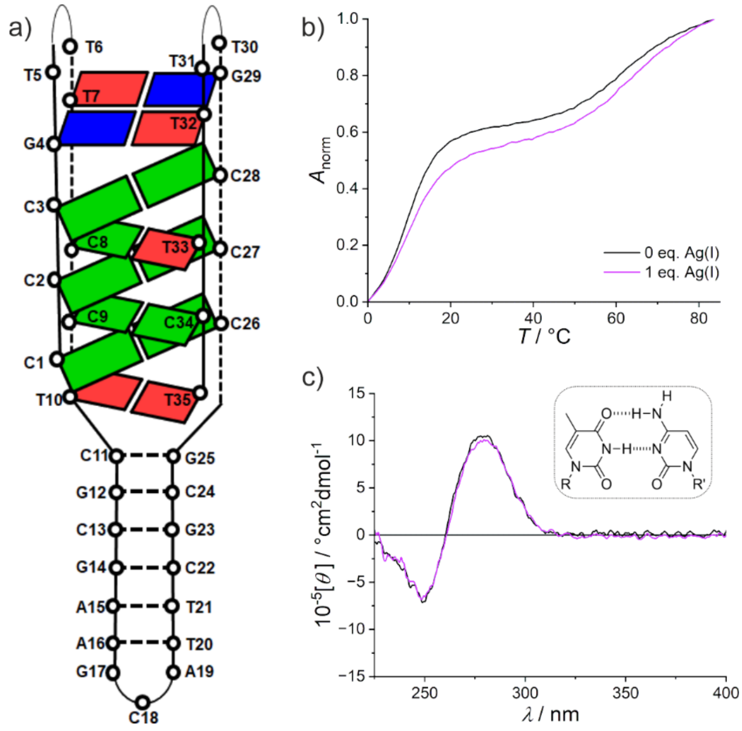

Figure S7. a) Schematic representation of IDJ1_T33. Selected nucleobases are depicted as colored rectangles (C, green; T, red; G, blue). b) Melting curves and c) CD spectra of IDJ1_T33 in the presence of increasing amounts of Ag^{I}. The inset shows the structure of a T:C mismatch. Conditions: 1 μM IDJ1_T33, 50 mM $NaClO_4$, 25 mM MOPS (pH 7.0).

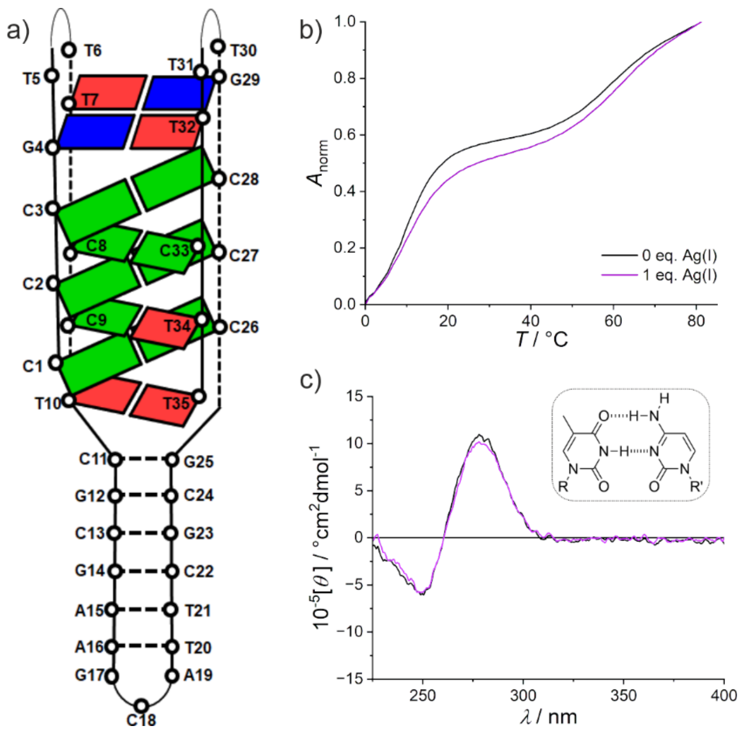

Figure S8. a) Schematic representation of IDJ1_T34. Selected nucleobases are depicted as colored rectangles (C, green; T, red; G, blue). b) Melting curves and c) CD spectra of IDJ1_T34 in the presence of increasing amounts of Ag^{I}. The inset shows the structure of a T:C mismatch. Conditions: 1 μM IDJ1_T34, 50 mM $NaClO_4$, 25 mM MOPS (pH 7.0).

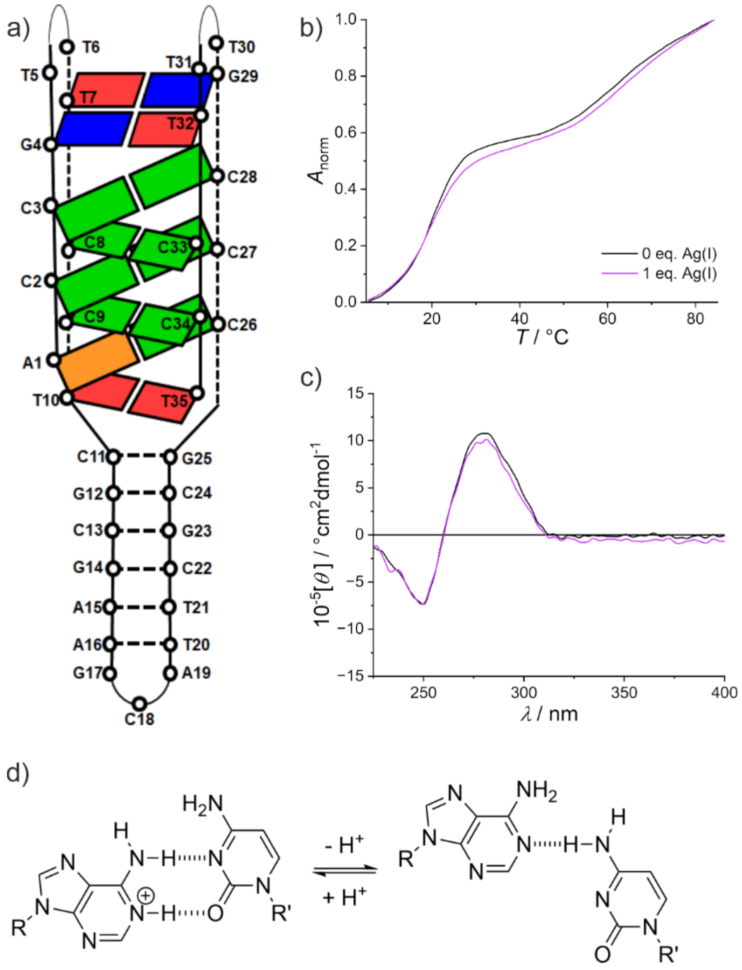

Figure S9. a) Schematic representation of IDJ1_A1. Selected nucleobases are depicted as colored rectangles (C, green; T, red; G, blue; A, orange). b) Melting curves and c) CD spectra of IDJ1_A1 in the presence of increasing amounts of Ag^{I}. d) pH-dependent structure of the A:C mismatch.[2] Conditions: 1 µM IDJ1_A1, 50 mM $NaClO_4$, 25 mM MOPS (pH 7.0).

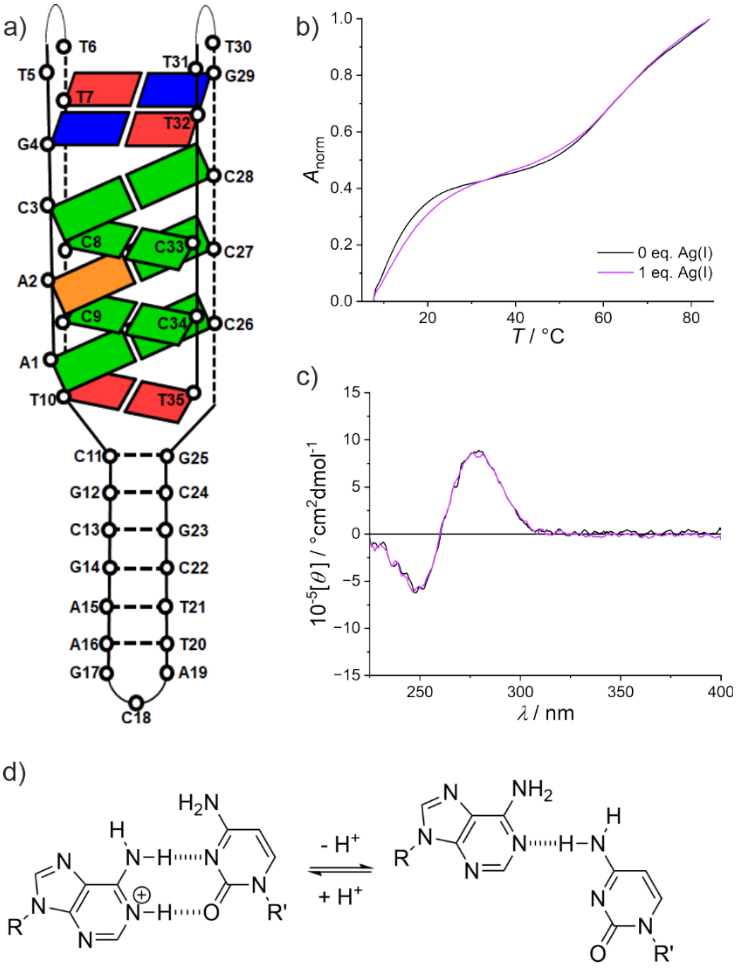

Figure S10. a) Schematic representation of IDJ1_A2. Selected nucleobases are depicted as colored rectangles (C, green; T, red; G, blue; A, orange). b) Melting curves and c) CD spectra of IDJ1_A2 in the presence of increasing amounts of Ag^{I}. d) pH-dependent structure of the A:C mismatch.[2] Conditions: 1 µM IDJ1_A2, 50 mM $NaClO_4$, 25 mM MOPS (pH 7.0).

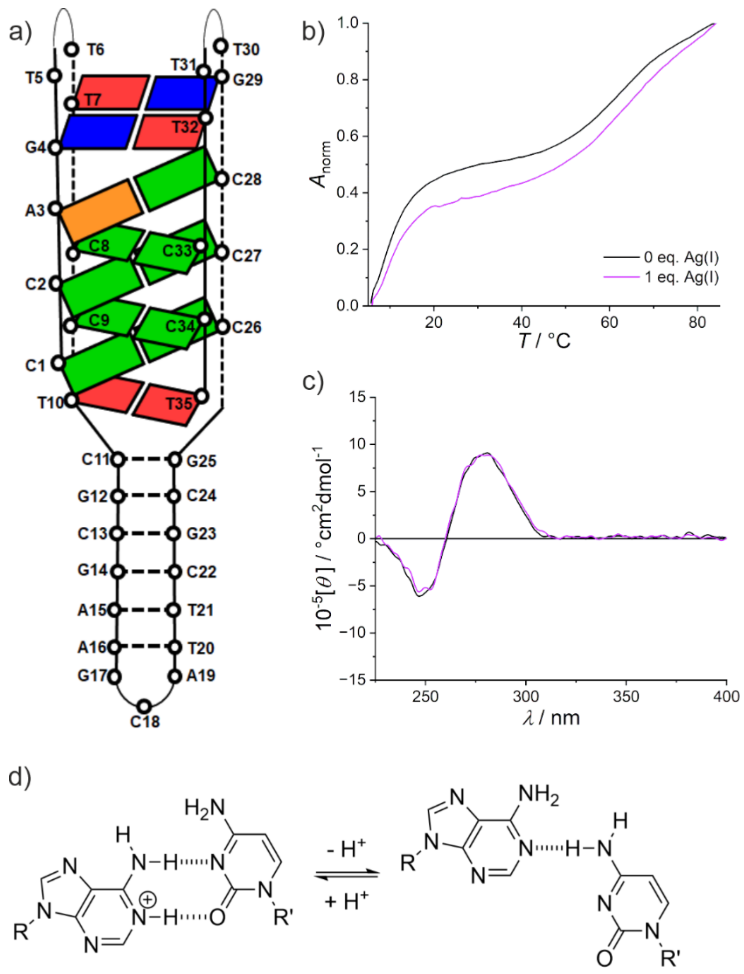

Figure S11. a) Schematic representation of IDJ1_A3. Selected nucleobases are depicted as colored rectangles (C, green; T, red; G, blue; A, orange). b) Melting curves and c) CD spectra of IDJ1_A3 in the presence of increasing amounts of Ag^{I}. d) pH-dependent structure of the A:C mismatch.[2] Conditions: 1 μM IDJ1_A3, 50 mM $NaClO_4$, 25 mM MOPS (pH 7.0).

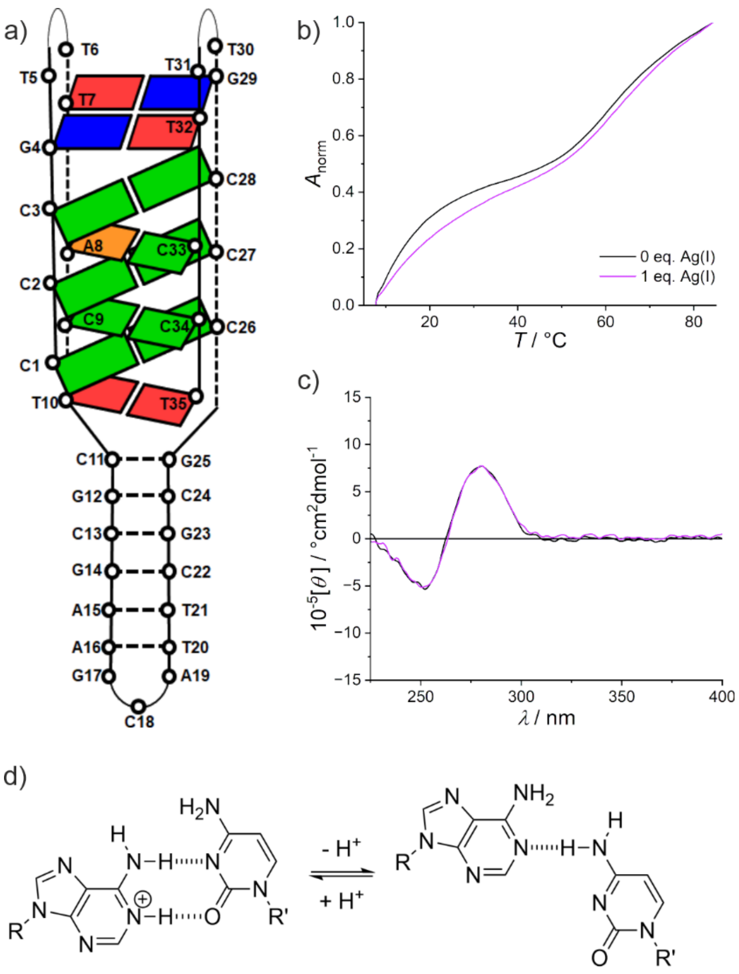

Figure S12. a) Schematic representation of IDJ1_A8. Selected nucleobases are depicted as colored rectangles (C, green; T, red; G, blue; A, orange). b) Melting curves and c) CD spectra of IDJ1_A8 in the presence of increasing amounts of Ag^{I}. d) pH-dependent structure of the A:C mismatch.[2] Conditions: 1 μM IDJ1_A8, 50 mM $NaClO_4$, 25 mM MOPS (pH 7.0).

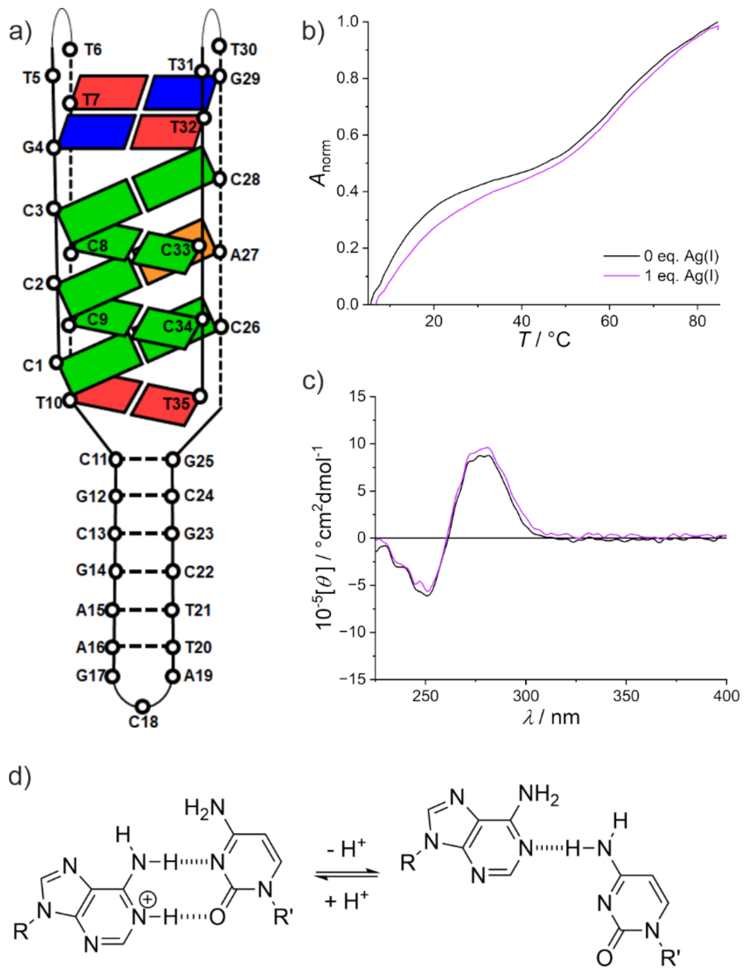

Figure S13. a) Schematic representation of IDJ1_A27. Selected nucleobases are depicted as colored rectangles (C, green; T, red; G, blue; A, orange). b) Melting curves and c) CD spectra of IDJ1_A27 in the presence of increasing amounts of Ag^{I}. d) pH-dependent structure of the A:C mismatch.[2] Conditions: 1 µM IDJ1_A27, 50 mM $NaClO_4$, 25 mM MOPS (pH 7.0).

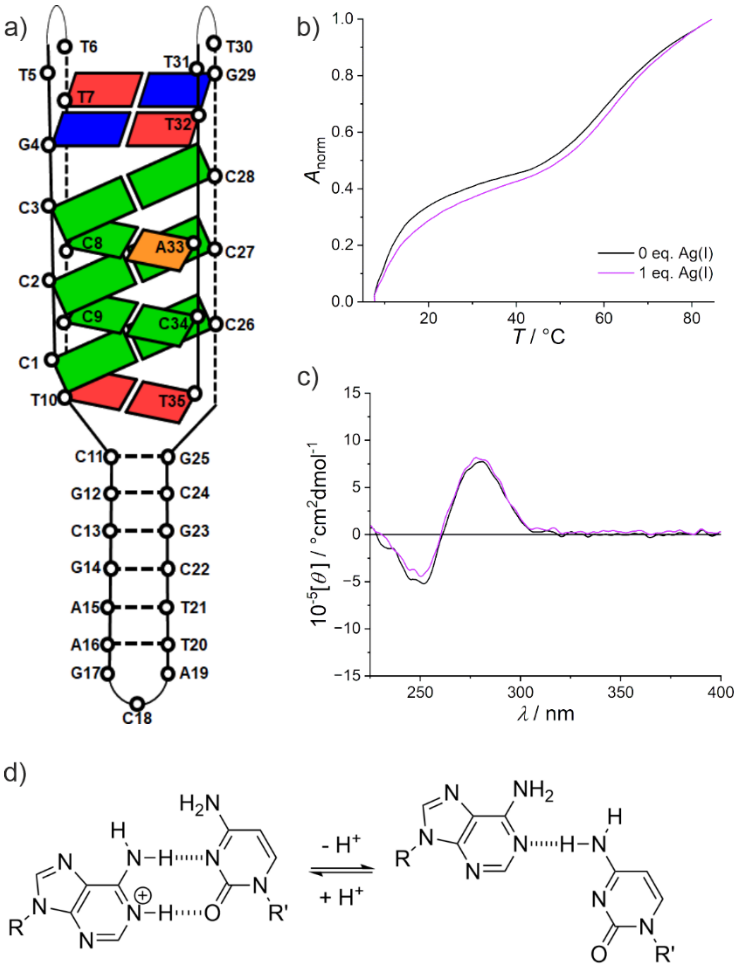

Figure S14. a) Schematic representation of IDJ1_A33. Selected nucleobases are depicted as colored rectangles (C, green; T, red; G, blue; A, orange). b) Melting curves and c) CD spectra of IDJ1_A33 in the presence of increasing amounts of Ag^{I}. d) pH-dependent structure of the A:C mismatch.[2] Conditions: 1 µM IDJ1_A33, 50 mM $NaClO_4$, 25 mM MOPS (pH 7.0).

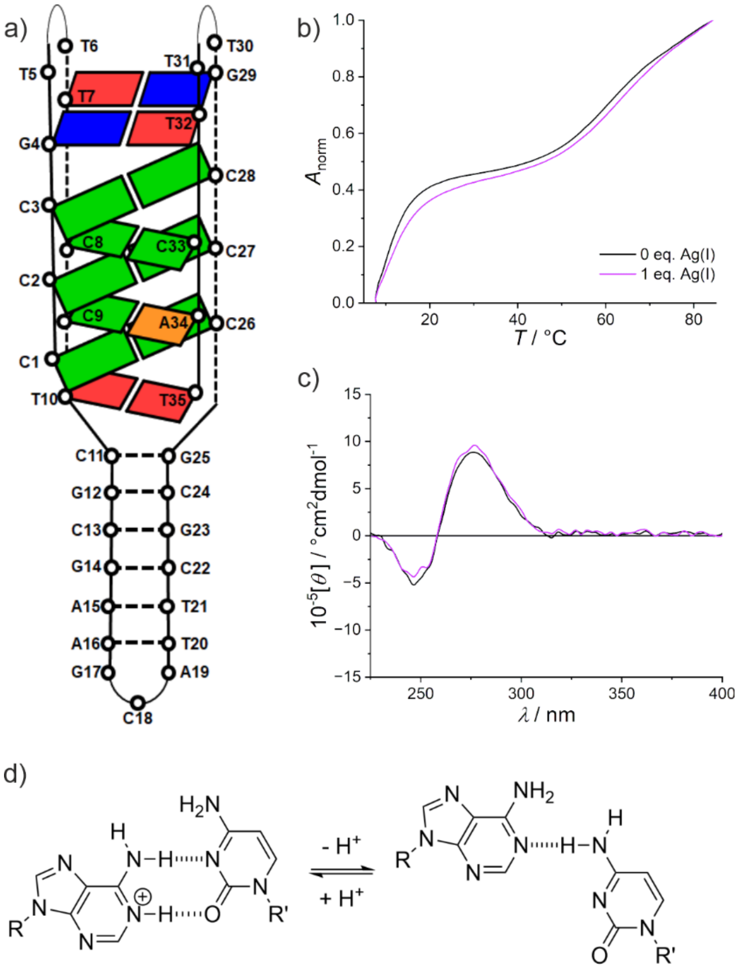

Figure S15. a) Schematic representation of IDJ1_A34. Selected nucleobases are depicted as colored rectangles (C, green; T, red; G, blue; A, orange). b) Melting curves and c) CD spectra of IDJ1_A34 in the presence of increasing amounts of Ag^{I}. d) pH-dependent structure of the A:C mismatch.[2] Conditions: 1 µM IDJ1_A34, 50 mM $NaClO_4$, 25 mM MOPS (pH 7.0).

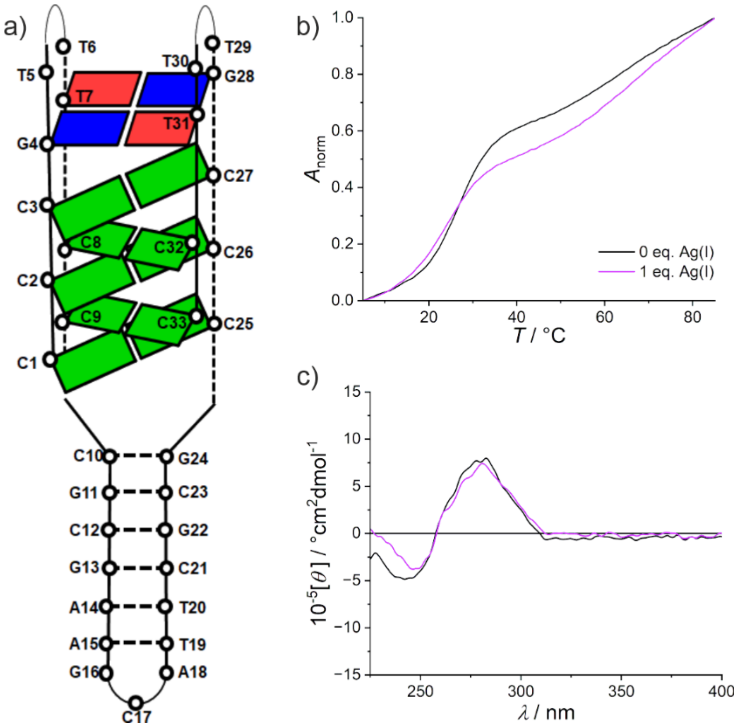

Figure S16. a) Schematic representation of IDJ2. Selected nucleobases are depicted as colored rectangles (C, green; T, red; G, blue). b) Melting curves and c) CD spectra of IDJ2 in the presence of increasing amounts of Ag^I. Conditions: 1 µM IDJ2, 50 mM $NaClO_4$, 25 mM MOPS (pH 7.0).

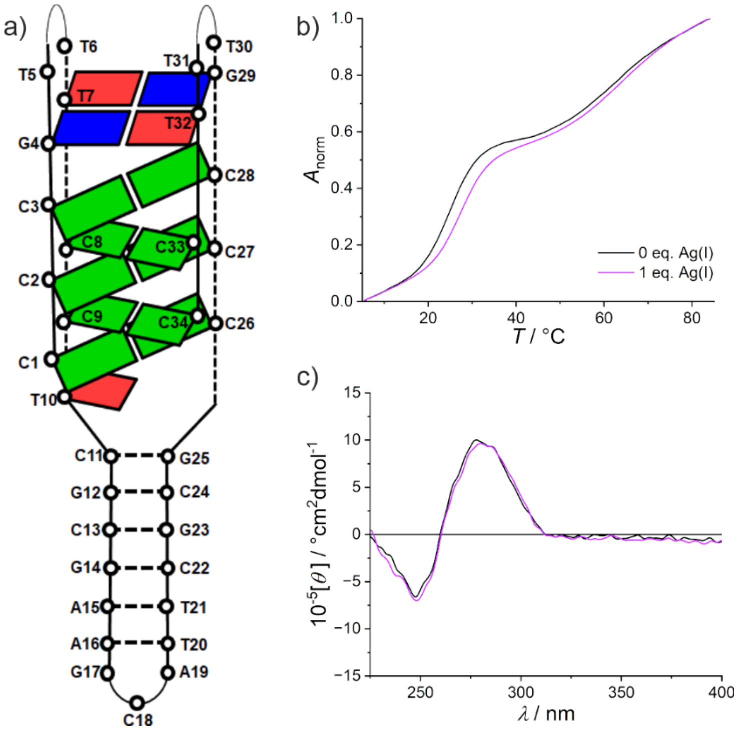

Figure S17. a) Schematic representation of IDJ3. Selected nucleobases are depicted as colored rectangles (C, green; T, red; G, blue). b) Melting curves and c) CD spectra of IDJ3 in the presence of increasing amounts of Ag^I. Conditions: 1 µM IDJ3, 50 mM $NaClO_4$, 25 mM MOPS (pH 7.0).

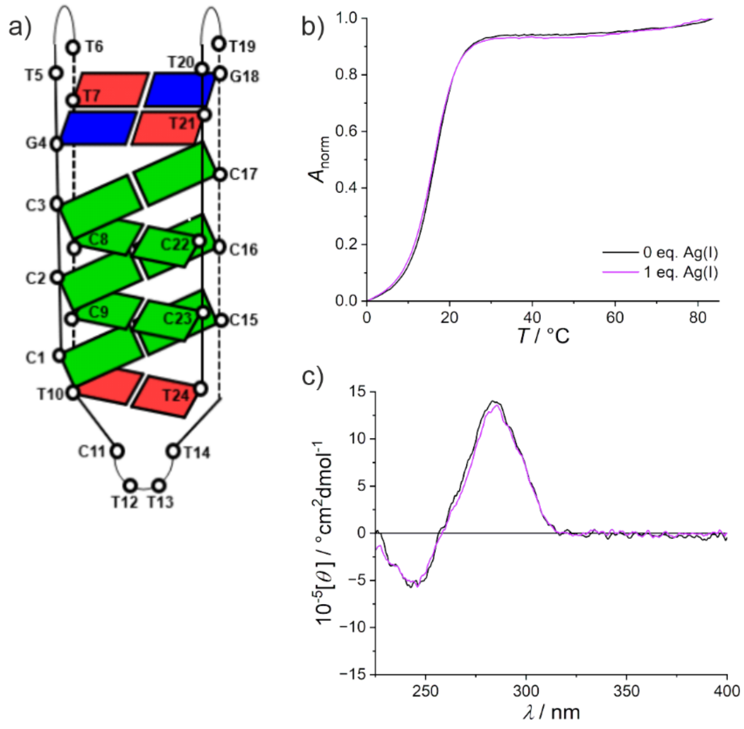

Figure S18. a) Schematic representation of IDJ4. Selected nucleobases are depicted as colored rectangles (C, green; T, red; G, blue). b) Melting curves and c) CD spectra of IDJ4 in the presence of increasing amounts of Ag^{I}. Conditions: 1 µM IDJ4, 50 mM $NaClO_4$, 25 mM MOPS (pH 7.0).

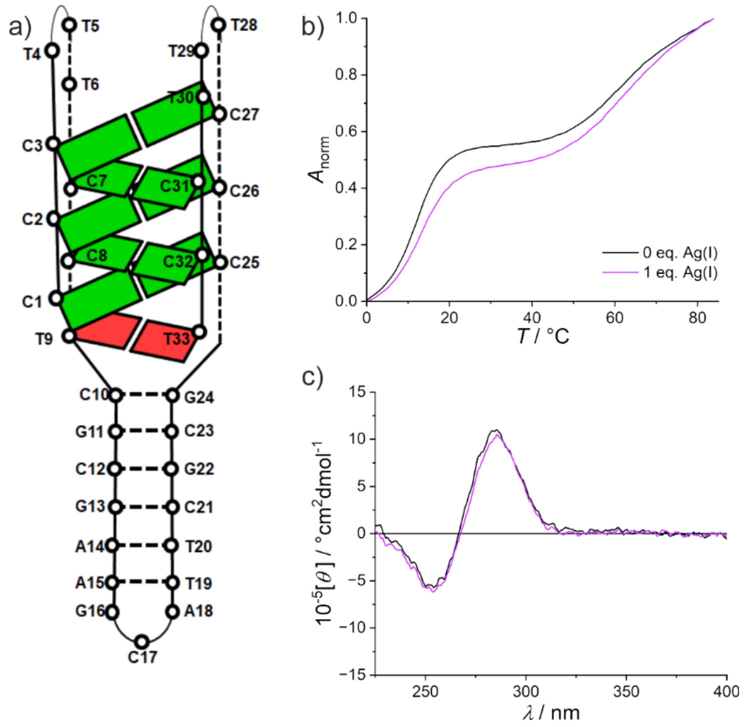

Figure S19. a) Schematic representation of IDJ5. Selected nucleobases are depicted as colored rectangles (C, green; T, red; G, blue). b) Melting curves and c) CD spectra of IDJ5 in the presence of increasing amounts of Ag^{I}. Conditions: 1 µM IDJ5, 50 mM $NaClO_4$, 25 mM MOPS (pH 7.0).

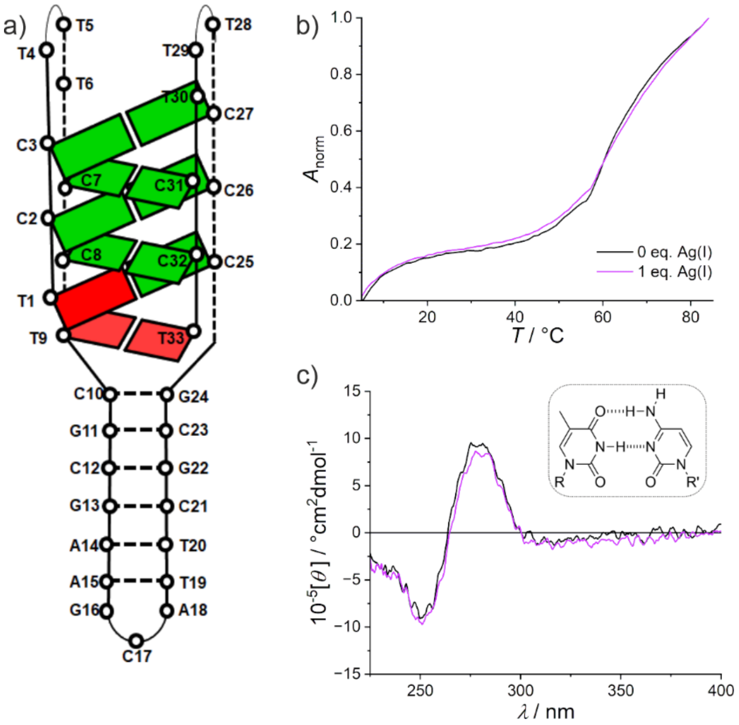

Figure S20. a) Schematic representation of IDJ5_T1. Selected nucleobases are depicted as colored rectangles (C, green; T, red; G, blue). b) Melting curves and c) CD spectra of IDJ5_T1 in the presence of increasing amounts of Ag^{I}. The inset shows the structure of a T:C mismatch. Conditions: 1 μM IDJ5_T1, 50 mM $NaClO_4$, 25 mM MOPS (pH 7.0).

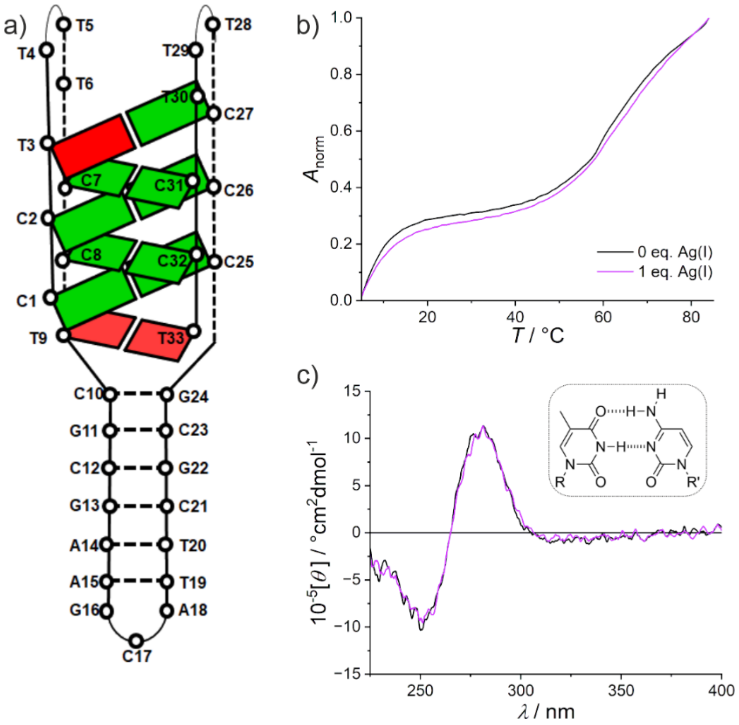

Figure S21. a) Schematic representation of IDJ5_T3. Selected nucleobases are depicted as colored rectangles (C, green; T, red; G, blue). b) Melting curves and c) CD spectra of IDJ5_T3 in the presence of increasing amounts of Ag^{I}. The inset shows the structure of a T:C mismatch. Conditions: 1 μM IDJ5_T3, 50 mM $NaClO_4$, 25 mM MOPS (pH 7.0).

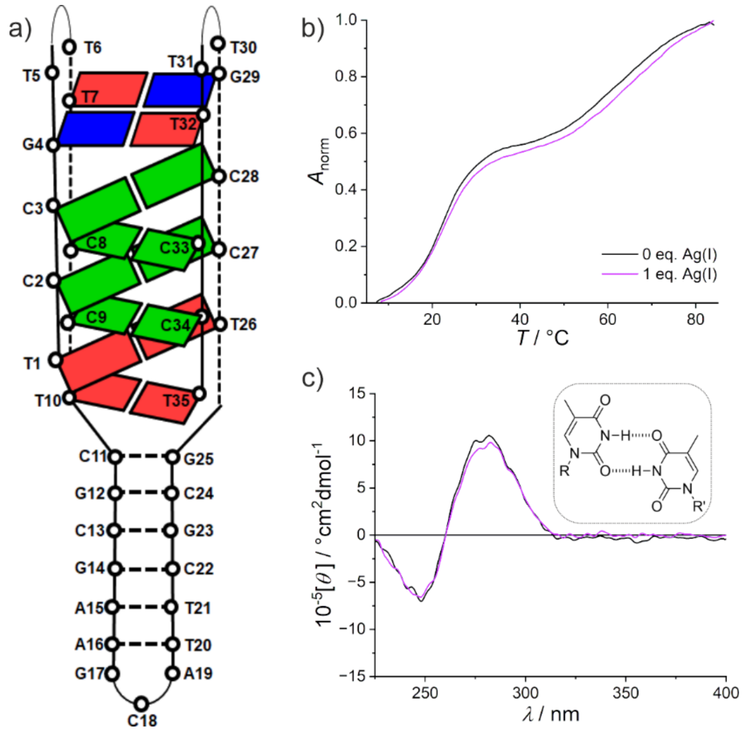

Figure S22. a) Schematic representation of IDJ6. Selected nucleobases are depicted as colored rectangles (C, green; T, red; G, blue). b) Melting curves and c) CD spectra of IDJ6 in the presence of increasing amounts of Ag^{I}. The inset shows the structure of a T:T mismatch. Conditions: 1 μM IDJ6, 50 mM $NaClO_4$, 25 mM MOPS (pH 7.0).

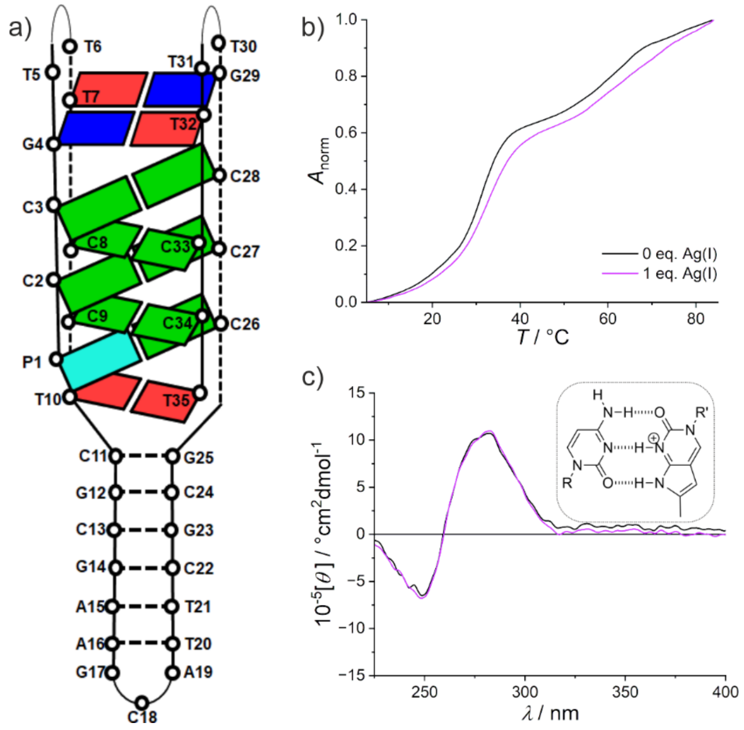

Figure S23. a) Schematic representation of IDJ1_P1. Selected nucleobases are depicted as colored rectangles (C, green; T, red; G, blue; P, light blue). b) Melting curves and c) CD spectra of IDJ1_P1 in the presence of increasing amounts of Ag^{I}. The inset shows the structure of a hemi-protonated C:PH^{+} pair. Conditions: 1 μM IDJ1_P1, 50 mM $NaClO_4$, 25 mM MOPS (pH 7.0).

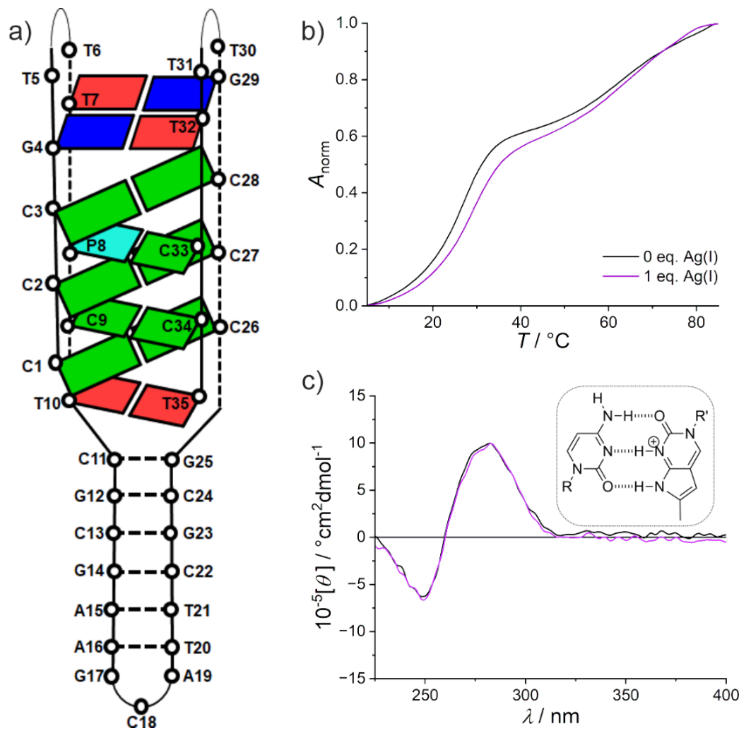

Figure S24. a) Schematic representation of IDJ1_P8. Selected nucleobases are depicted as colored rectangles (C, green; T, red; G, blue; P, light blue). b) Melting curves and c) CD spectra of IDJ1_P8 in the presence of increasing amounts of Ag^{I}. The inset shows the structure of a hemi-protonated C:PH^+ pair. Conditions: 1 µM IDJ1_P8, 50 mM $NaClO_4$, 25 mM MOPS (pH 7.0).

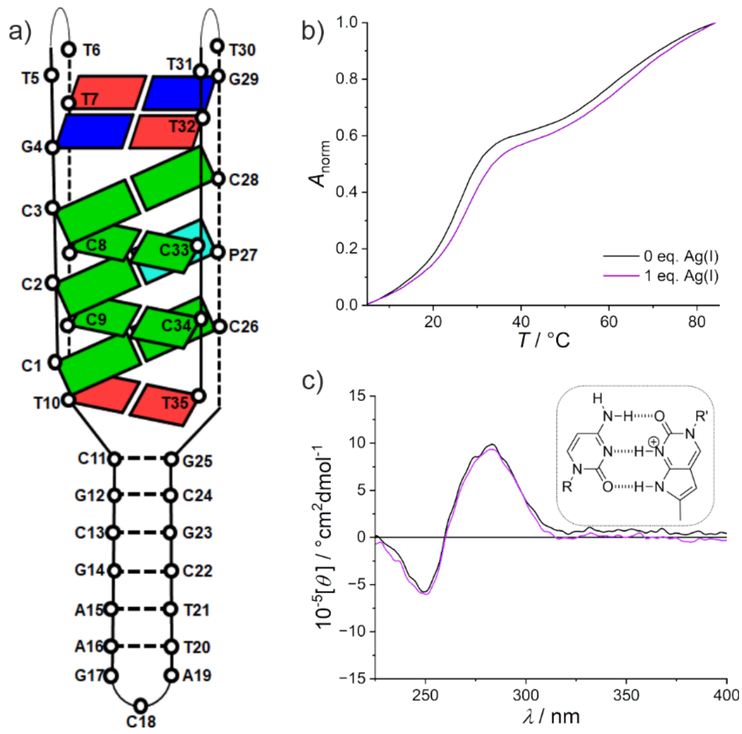

Figure S25. a) Schematic representation of IDJ1_P27. Selected nucleobases are depicted as colored rectangles (C, green; T, red; G, blue; P, light blue). b) Melting curves and c) CD spectra of IDJ1_P27 in the presence of increasing amounts of Ag^{I}. The inset shows the structure of a hemi-protonated C:PH^+ pair. Conditions: 1 µM IDJ1_P27, 50 mM $NaClO_4$, 25 mM MOPS (pH 7.0).

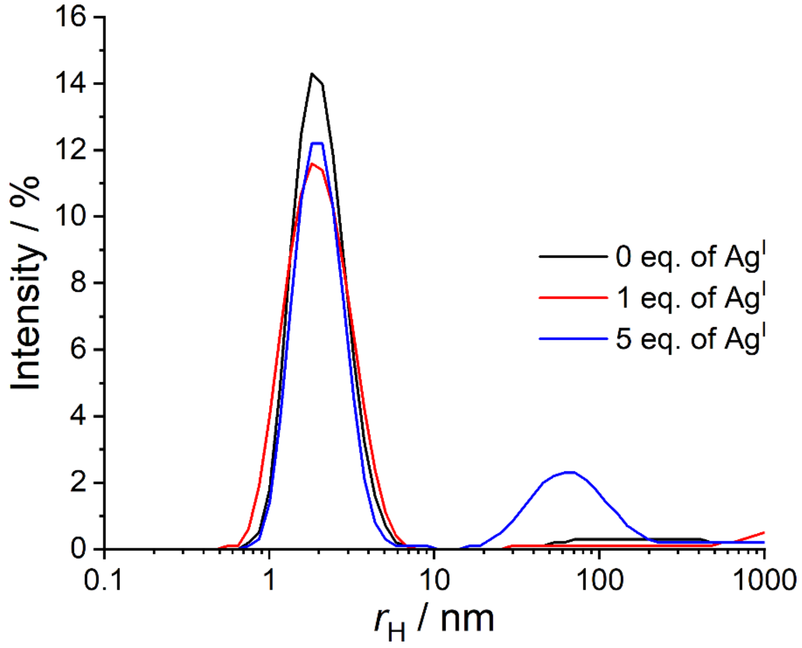

Figure S26. Intensity plots of the DSL measurements of IDJ1 in the presence of a) no Ag^I (black line), b) 1 eq. of Ag^I (red line), and c) 5 eq. of Ag^I (blue line). Conditions: 250 μM IDJ1, 50 mM $NaClO_4$, 25 mM MOPS (pH 7.0).

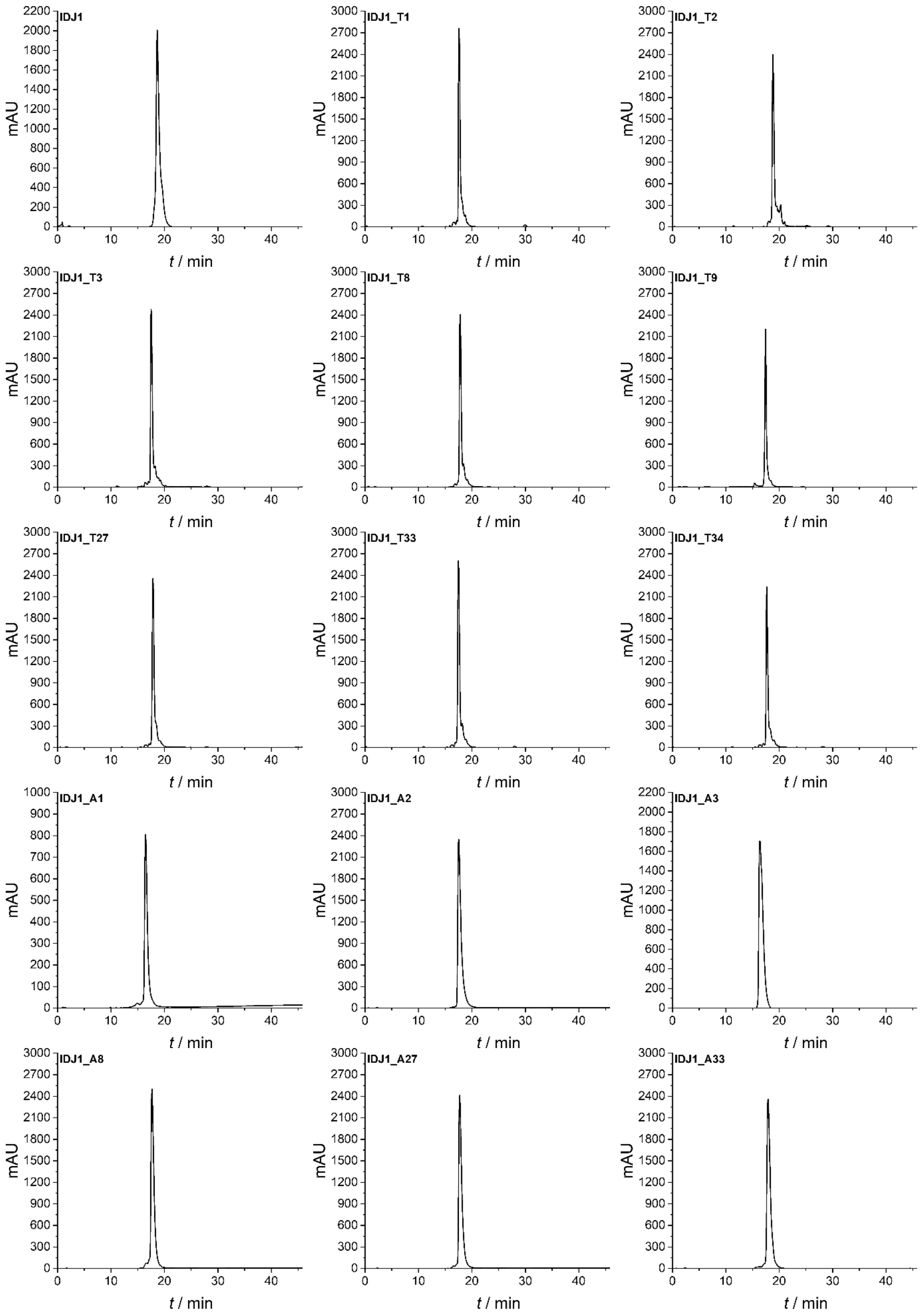

Figure S27. HPLC chromatograms of the oligonucleotides (purity control).

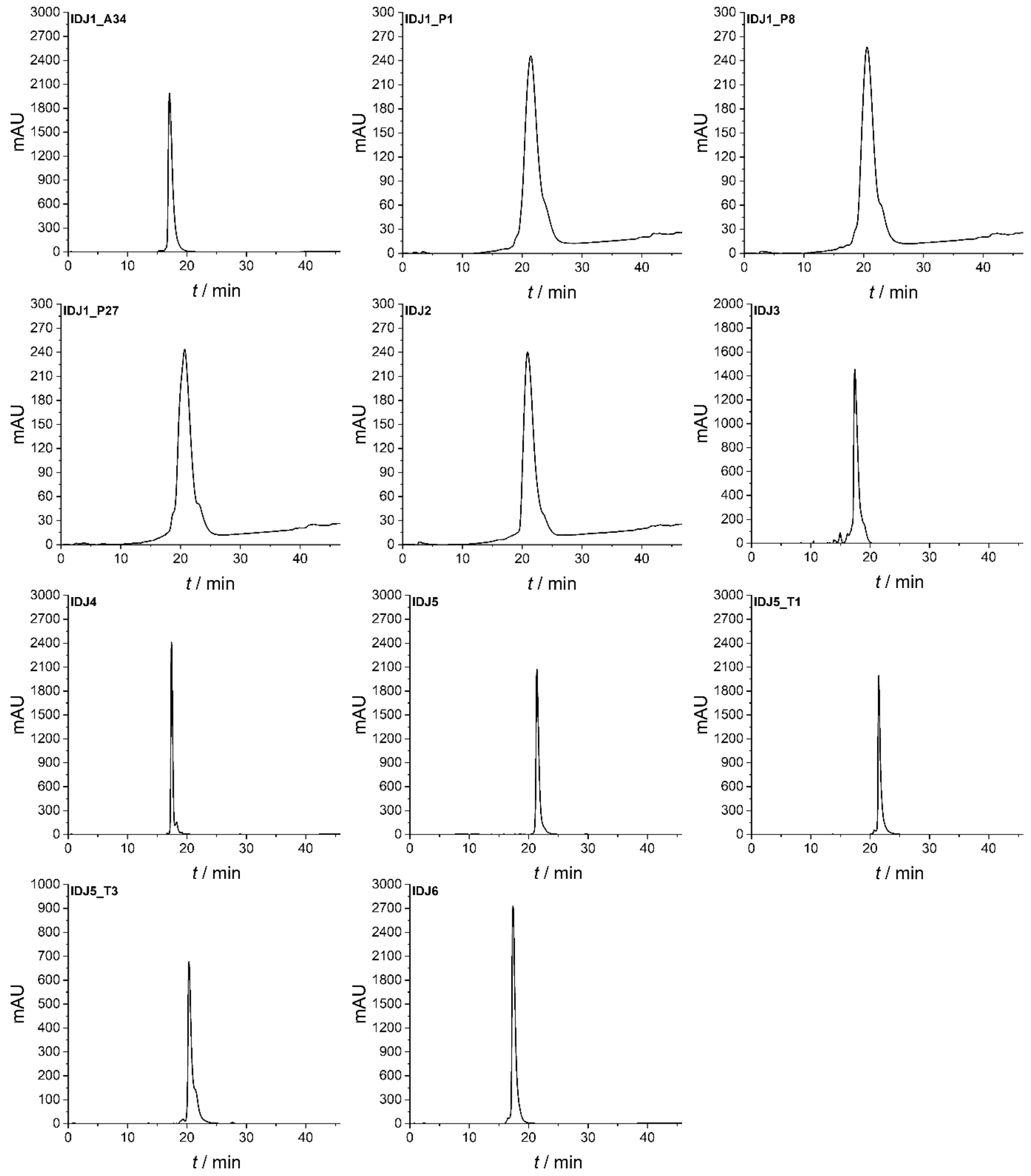

Figure S27 (continued). HPLC chromatograms of the oligonucleotides (purity control).

Table S1. Melting temperatures $T_{m,1}$ of the i-motif part of the IDJs in the presence of various amounts of Ag^I and increase in melting temperature $\Delta T_{m,1}$ upon the addition of one equivalent of Ag^I. [a]

Oligonucleotide	$T_{m,1}$ / °C (0 equiv.)	$T_{m,1}$ / °C (1 equiv.)	$\Delta T_{m,1}$ / °C (0→1 equiv.)
IDJ1	28.5 ± 0.1	31.7 ± 0.2	3.2 ± 0.2
IDJ1_T1	20.4 ± 0.4	20.0 ± 0.4	–0.4 ± 0.5
IDJ1_T2	12.8 ± 0.4	15.9 ± 0.5	3.1 ± 0.6
IDJ1_T3	9.2 ± 0.3	11.6 ± 0.3	2.4 ± 0.5
IDJ1_T8	12.9 ± 0.3	15.6 ± 0.4	2.7 ± 0.5
IDJ1_T9	11.9 ± 0.2	15.2 ± 0.3	3.3 ± 0.3
IDJ1_T27	7.3 ± 0.2	8.0 ± 0.3	0.7 ± 0.4
IDJ1_T33	9.1 ± 0.3	9.4 ± 0.5	0.3 ± 0.6
IDJ1_T34	10.1 ± 0.4	9.8 ± 0.4	–0.3 ± 0.6
IDJ1_A1	19.9 ± 0.4	19.7 ± 0.3	–0.2 ± 0.4
IDJ1_A2	8 ± 2	10 ± 1	2 ± 2
IDJ1_A3	8 ± 2	10 ± 1	2 ± 2
IDJ1_A8	1 ± 8	[a]	[b]
IDJ1_A27	3 ± 6	6 ± 6	3 ± 9
IDJ1_A33	1 ± 12	[a]	[b]
IDJ1_A34	9 ± 1	9 ± 1	1 ± 2
IDJ1_P1	31.1 ± 0.4	32.3 ± 0.5	1.1 ± 0.7
IDJ1_P8	26.2 ± 0.5	27.3 ± 0.4	1.1 ± 0.6
IDJ1_P27	25.4 ± 0.5	27.3 ± 0.4	1.9 ± 0.7
IDJ2	26.7 ± 0.2	23.9 ± 0.4	–2.8 ± 0.4
IDJ3	24.6 ± 0.3	27.2 ± 0.2	2.6 ± 0.4
IDJ4	19.9 ± 0.4	19.7 ± 0.3	–0.2 ± 0.4
IDJ5	11.8 ± 0.3	12.8 ± 0.3	1.1 ± 0.4
IDJ5_T1	[a]	[a]	[b]
IDJ5_T3	[a]	[a]	[b]
IDJ6	22.3 ± 0.4	22.4 ± 0.2	0.1 ± 0.5

[a] fit resulted in unrealistic value. [b] not determined.

Experimental Details

DNA synthesis

The oligonucleotides were synthesized by solid-phase DNA synthesis using an H-6 or an H-8 DNA/RNA Synthesizer by *K&A Laborgeräte* in 'DMT-ON' mode following the standard protocol. All phosphoramidites were purchased from *Glen Research*. The oligonucleotides were cleaved off the solid support and the protection groups were removed by using methylamine/ammonia (1:1) at 65 °C for 15 min or ammonia (25 %) at 55 °C for 16 h. The oligonucleotides were purified using Glen-Pak cartridges according to manufacturer's protocol. Some oligonucleotides (IDJ1_A2, IDJ1_A3, IDJ1_A27, IDJ1_A33, IDJ1_A34, IDJ3, IDJ6) were additionally purified by RP-HPLC (Multi-wavelength Detector MD-2010 PLUS, HPLC Pump PU-2080 PLUS, Gradient Unit LG-2080-02, Degasser DG-2080-53) from *Jasco* equipped with a *Nucleodur* column (C18 HTec, particle size 5 µm, dimensions: 125 mm × 4 mm) from *Macherey-Nagel*. The buffer gradients between buffer A (10 mM triethylammonium acetate (TEAA) in water, pH 7.0) and buffer B (10 mM TEAA in water/acetonitrile (20/80)) are displayed in Table S2. The flow rate was set to 1 mL min^{-1}.

Table S2. Timeline of buffer composition during purification.

t / min	0	5	25	40	46
Buffer A / %	100	97	85	20	0
Buffer B / %	0	3	15	80	100

Purification control

The purity of the oligonucleotides was verified by RP-HPLC using the setup mentioned above. The buffer gradient is displayed in Table S3. The chromatograms are depicted in Figure S27.

Table S3. Timeline of buffer composition during purification.

t / min	0	5	45	50	61
Buffer A / %	100	97	60	20	0
Buffer B / %	0	3	40	80	100

Concentration determination of DNA strands

The concentrations of DNA strands were determined using a *Nanodrop 200c Photometer* (*Thermo Scientific*) by measuring their absorbance at 260 nm.

Sample preparation

The samples for the spectroscopic characterization contained 1 μM IDJ dissolved in 50 mM $NaClO_4$, 25 mM MOPS buffer (pH 7.0). After the addition of $AgNO_3$, the samples were annealed from 85 °C to 5 °C with a cooling rate of 1 °C min^{-1}. Samples for the mass-spectrometric characterization were prepared by combining 5 μM IDJ, 50 mM $NaClO_4$ and 25 mM MOPS buffer (pH 7.0). A stochiometric amount of $AgNO_3$ was added and the sample was heated to 85 °C and cooled back to 10 °C at a rate of 1 °C min^{-1}. The solution was shock-frosted using liquid N_2, lyophilized, and re-dissolved in water (50 μL).

DNA melting experiments

The temperature-dependent UV spectra for the DNA melting studies were recorded on a *Jasco V-750 spectrometer*. A quartz cuvette with 1 cm path length was used. Temperature-dependent UV absorbance at 260 nm was recorded between 5 °C and 85 °C with a heating/cooling rate of 1 °C min^{-1} and a data interval of 1 °C. Absorbance was normalized according to $A_{norm} = (A - A_{min}) / (A_{max} - A_{min})$. The melting curves were smoothed with the *Adjacent-Averaging* filter in steps of 5 using *OriginPro 2021*. The melting points were determined from the maxima of the first derivative of the melting curves.

CD spectroscopy

CD spectra were recorded at 5 °C from 225 to 400 nm using a *Jasco 815* CD spectrometer. The quartz cuvette had a path length of 1 cm. For the baseline correction, CD spectra of the respective solution without DNA were recorded and were subtracted from the CD spectra of the samples. Furthermore, the CD spectra were smoothed with the *Adjacent-Averaging* filter in steps of 25 using *OriginPro 2021*.

Fluorescence spectroscopy

Fluorescence spectra were recorded on a *Jasco FP-8300* at 5 °C from 370 to 700 nm using an excitation wavelength of 350 nm. The fluorescence spectra were corrected by taking into account the inner filter effect (IFE).[4] Afterwards, emission was normalized according to $I_{norm} = (I_{IFE} - I_{IFE,min}) / (I_{IFE,max} - I_{IFE,min})$ (Figure 6, top row) and $I_{norm} = (I_{IFE}/I_{IFE,\,max}) \cdot 100$ (Figure 6, bottom row), respectively.

LC-MS

For the mass spectrometric characterization of the C–Ag^{I}–C base pair in the i-motif, a timsToF flex instrument (Bruker Daltonik) was employed in combination with an upstream UltiMate 3000 UHPLC system from Thermo Fisher Scientific. The mobile phase consisted of an aqueous 50 mM NH_4OAc solution, and the flow rate was set at 0.4 mL min^{-1}. The spectrum was recorded in negative ion mode.

Determination of the hydrodynamic radius

The size of IDJ1 was estimated based on the reported solution structure.[1] Considering an average distance between consecutive base pairs of 0.31 nm for the i-motif and 0.34 nm for the B-DNA part of IDJ1,[3] a length L of 4 × 0.31 nm + 7 × 0.34 nm + 1 nm (hairpin loop) + 1 nm (i-motif loop) = 5.62 nm results. The average diameter d amounts to 2.5 nm. As q = L/q > 2, the following symmetrical cylinder model was applied:[4]

$$r_\mathrm{H} = \frac{L/2}{\ln q + 0.312 + 0.565\, q^{-1} - 0.1 q^{-2}}$$

Dynamic light scattering was performed using an Anton Paar Litesizer DLS501 instrument with a 50 µL quartz cuvette. Measurements were carried out at a scattering angle of 90°, using oligonucleotide concentrations of 250 µM in a buffer containing 50 mM $NaClO_4$ and 25 mM MOPS (pH 7). Prior to the measurements, all samples were centrifuged at 14,000 rpm for 30 min. For the samples containing Ag^I, 1 or 5 equivalents of $AgNO_3$ were, followed by heating the solution to approximately 70 °C and then slowly cooling it back to 25 °C at a rate of 3 °C min^{-1}. Intensity plots were evaluated in the determination of r_H.

^{1}H NMR spectroscopy of IDJ1

NMR spectra were recorded on a Bruker Avance NEO 600 spectrometer using an oligonucleotide concentration of 500 µM in a buffer containing 9:1 H_2O/D_2O, 50 mM $NaClO_4$, 25 mM MOPS (pH 7) and 50 ppm TSP (as internal reference). The measurements were performed at 25 °C.

Isothermal titration calorimetry

ITC (isothermal titration calorimetry) experiments were carried out using a Nano-ITC low volume instrument (169 µL cell volume) (TA Instruments), while fitting was made using the software included with the calorimeter (NanoAnalyze Data Analysis Software 4.1.0, TA Instruments). The calorimeter was stabilized at 25 °C for 1 h prior to the measurements. All solutions were buffered in 25 mM MOPS buffer at pH 7.0 containing 50 mM $NaClO_4$. It was important to keep the buffer conditions constant during the experiment. All solutions containing $AgNO_3$ were protected from light to avoid degradation.

The cells were filled with an excess of 300 µL of a ca. 150 µM DNA duplex solution (working cell) and 300 µL of ultra-pure water (reference cell) to avoid air bubbles. The 52 µL titration syringe was loaded with 52 µL of a ca. 2, 3 or 5 mM of $AgNO_3$ solution. The calorimeter was stabilized for 30 min, for expected low-heat measurements. The experiments were set to 40 injections of 1.28 µL with a collecting time of 300 s between injections and a stirring rate of 250 rpm and with 60 s of initial and final baseline measurement. Once measured, the raw heat was corrected for dilution effects by subtracting the dilution heat (control; injection of an $AgNO_3$ solution into the buffer under the same conditions as the regular experiment). All experiments were performed in triplicate.

Based on the data reported in the accompanying manuscript, i-motifs cannot accommodate more than one C–Ag^I–C base pair and duplexes are formed instead. It is therefore likely that previously reported i-motifs with C–Ag^I–C base pairs are in fact duplexes containing these base pairs. In fact, the experimental data from those previous reports are compatible with duplex formation. The following section presents alternative explanations of the respective experimental data, in line with the formation of duplexes bearing C–Ag^I–C base pairs. In those cases where applications were reported for the "silver(I)-containing i-motif", the alternative explanation does not invalidate the reported applications, it merely presents a different interpretation of the underlying nucleic acid structure.

Alternative explanation of the data from Chem. Commun. 2013, 49, 7696

The increase in melting temperature upon the addition of Ag^I (Fig. S2) does not necessarily indicate the formation of C–Ag^I–C base pairs within the i-motif, it could likewise be explained by the formation of these base pairs within a double helix.

The changes in the CD spectrum upon the addition of Ag^I (Fig. 2), i.e., the red-shift of both the positive and the negative Cotton effects with decreasing molar ellipticity are likely to show the structural rearrangement towards a double helix with C–Ag^I–C base pairs (rather than an incorporation of Ag^I ions into the i-motif). The final CD spectrum closely resembles that of such a double helix.[5-7]

The 4:1 binding stoichiometry (Ag^I ions per oligonucleotide) determined by Job plot analysis (Fig. S9) can also be explained by formation of a double helix containing eight Ag^I-mediated base pairs. In fact, previous studies have shown that oligonucleotides designed to form duplexes with a certain number of regular Watson-Crick base pairs can rearrange into duplexes with fewer Ag^I-mediated base pairs,[7-8] which could explain why a stoichiometry is observed that is less what than would be expected if all C–Ag^I–C positions were occupied.

The FRET-based determination of oligonucleotide folding upon the addition of Ag^I (Fig. 3) and the corresponding unfolding upon the addition of cysteine (Fig. S14) are in good agreement with the formation of a double helix containing C–Ag^I–C base pairs, as this duplex also brings the fluorophores into close proximity of each other.

Alternative explanation of the data from Chem. Commun. 2014, 50, 15385

The fluorescence of the cyanine dye DMSB is used to signal the formation of an i-motif structure. Reference measurements on the fluorescence of DMSB in a presence of a duplex composed of Ag^I-mediated base pairs are not reported. Hence, it is not clear whether the fluorescence increase of DMSB in the presence of cAS1411 and Ag^I could not also be due to the formation of a double helix with C–Ag^I–C base pairs.

Alternative explanation of the data from ACS Sens. 2020, 5, 2177

The changes in the CD spectrum upon the addition of Ag^I (Fig. S3), i.e., the red-shift of both the positive and the negative Cotton effects with decreasing molar ellipticity are likely to show the structural rearrangement towards a double helix with C–Ag^I–C base pairs (rather than an incorporation of Ag^I ions into the i-motif).

While there is no doubt that the oligonucleotide undergoes a structural change upon the addition of Ag^I, this change is not necessarily a transition to an i-motif. The formation of an intramolecular hairpin structure with C–Ag^I–C base pairs is more likely. In fact, when the authors state that "Ag^+ formed C−Ag^+−C mismatch with cytosine after $AgNO_3$ was added to the solution, which led to ssDNA being folded into a rigid quadruplex i-motif structure.[29]" (page 2179), they refer to "reference 29", in which the formation of a hairpin structure is proposed (rather than an i-motif).[9]

The fluorescence intercalator displacement assay, showing a displacement of MB upon the binding of Ag^I to the DNA, is in good agreement with the formation of a (hairpin) duplex containing C–Ag^I–C base pairs. The positively charged MB is not able to intercalate between these positively charged base pairs.

For the actual nanopore experiments, the essential conclusion seems to be that the presence of Ag^I leads to the formation of a rigid oligonucleotide structure, which is destroyed again upon the removal of Ag^I (by means of GSH). Hence, all conclusions remain valid when assuming the formation of a hairpin with C–Ag^I–C base pairs rather than of a Ag^I-stabilized i-motif.

Alternative explanation of the data from Angew. Chem. Int. Ed. 2024, 63, e202407838

The changes in the CD spectrum upon the addition of Ag^I (Fig. S1a, Fig. 2c), i.e., a red-shift of the negative Cotton effect and a generally decreasing molar ellipticity are compatible with a structural rearrangement towards a double helix with C–Ag^I–C base pairs (rather than an incorporation of Ag^I ions into the i-motif).[5-7] In fact, the authors themselves state that "the helicity of ODN-**1**(Ag^+) is different with ODN-**1** containing C:C^+ base pairs" (page 4). The formation of a duplex rather than an i-motif could also explain why the catalytic activity in the presence of Cu^{II} was much better for the Ag^I-containing oligonucleotide compared to the proton-stabilized i-motif, namely because of different nucleic acid topologies.

The observation that ODN-**1** binds six to seven Ag^I ions (based on ESI-MS spectra, Fig. S2) could also relate to a non-specific binding of the Ag^I ions (for charge-screening reasons).

The disappearance of the C:CH^+ imino proton resonances in the ^1H NMR spectrum of ODN-**1** (Fig. 2e) is in good agreement with the destruction of the i-motif structure. It does not necessarily mean that the protons are replaced by Ag^I ions, it could also be explained by a rearrangement to a duplex with Ag^I-mediated base pairs. In fact, the observation that the resonances disappear once *one* Ag^I ion is present per ODN-**1** (even though ODN-**1** forms *six* C:CH^+ pairs in its i-motif topology) is in good agreement with a Ag^I-induced structural rearrangement away from the i-motif.

The increase in melting temperature upon the addition of Ag^I (Fig. S4) does not necessarily indicate the formation of C–Ag^I–C base pairs within the i-motif, it could likewise be explained by the formation of these base pairs within a double helix.

The stoichiometric value of six Ag^I ions per ODN-**1** as determined by ITC (Fig. 3) can likewise be explained by formation of a double helix containing twelve Ag^I-mediated base pairs. This is also supported by the negative entropy change, which would be expected upon the association of two single strands to form one double helix. Similarly, the binding of one Cu^{II} per ODN-**1** would be equivalent with the binding of two Cu^{II} ions per duplex, as the latter would contain two designated Cu^{II}-binding sites.

References

[1] I. Serrano-Chacón, B. Mir, N. Escaja, C. González, "Structure of i-Motif/Duplex Junctions at Neutral pH" *J. Am. Chem. Soc.* **2021**, *143*, 12919-12923.

[2] Y. Boulard, J. A. H. Cognet, J. Gabarro-Arpa, M. Le Bret, L. C. Sowers, G. V. Fazakerley, "The pH dependent configurations of the C.A mispair in DNA" *Nucleic Acids Res.* **1992**, *20*, 1933-1941.

[3] H. Abou Assi, M. Garavís, C. González, M. J. Damha, "i-Motif DNA: structural features and significance to cell biology" *Nucleic Acids Res.* **2018**, *46*, 8038-8056.

[4] J. Lapham, J. P. Rife, P. B. Moore, D. M. Crothers, "Measurement of diffusion constants for nucleic acids by NMR" *J. Biomol. NMR* **1997**, *10*, 255-262.

[5] S. M. Swasey, E. G. Gwinn, "Silver-mediated base pairings: towards dynamic DNA nanostructures with enhanced chemical and thermal stability" *New J. Phys.* **2016**, *18*, 045008.

[6] S. M. Swasey, L. Espinosa Leal, O. Lopez-Acevedo, J. Pavlovich, E. G. Gwinn, "Silver (I) as DNA glue: Ag^+-mediated guanine pairing revealed by removing Watson-Crick constraints" *Sci. Rep.* **2015**, *5*, 10163.

[7] T. Atsugi, A. Ono, M. Tasaka, N. Eguchi, S. Fujiwara, J. Kondo, "A Novel Ag^I-DNA Rod Comprising a One-Dimensional Array of 11 Silver Ions within a Double Helical Structure" *Angew. Chem. Int. Ed.* **2022**, *61*, e202204798.

[8] J. Kondo, Y. Tada, T. Dairaku, Y. Hattori, H. Saneyoshi, A. Ono, Y. Tanaka, "A metallo-DNA nanowire with uninterrupted one-dimensional silver array" *Nat. Chem.* **2017**, *9*, 956-960.

[9] L. Gao, P. Li, Y. Zhang, K. Xiao, J. Ma, G. Xie, G. Hou, Z. Zhang, L. Wen, L. Jiang, "A Bio-inspired, Sensitive, and Selective Ionic Gate Driven by Silver (I) Ions" *Small* **2015**, *11*, 543-547.
